# Clustered protocadherins methylation alterations in cancer

**DOI:** 10.1186/s13148-019-0695-0

**Published:** 2019-07-09

**Authors:** Ana Florencia Vega-Benedetti, Eleonora Loi, Loredana Moi, Sylvain Blois, Antonio Fadda, Manila Antonelli, Antonella Arcella, Manuela Badiali, Felice Giangaspero, Isabella Morra, Amedeo Columbano, Angelo Restivo, Luigi Zorcolo, Viviana Gismondi, Liliana Varesco, Sara Erika Bellomo, Silvia Giordano, Matteo Canale, Andrea Casadei-Gardini, Luca Faloppi, Marco Puzzoni, Mario Scartozzi, Pina Ziranu, Giuseppina Cabras, Pierluigi Cocco, Maria Grazia Ennas, Giannina Satta, Mariagrazia Zucca, Daniele Canzio, Patrizia Zavattari

**Affiliations:** 1 Department of Biomedical Sciences, Unit of Biology and Genetics, University of Cagliari, Cagliari, Italy; 2 Department of Radiological, Oncological and Anatomo-Pathological Sciences, University Sapienza of Rome, Rome, Italy; 3 IRCCS Neuromed, Pozzilli, Italy; 4 Genetic and Genomic Laboratory, Microcitemico Children’s Hospital, Cagliari, Italy; 5 Department of Pathology OIRM-S, Anna Hospital, A.O.U. City of Health and Science, Turin, Italy; 6 Department of Biomedical Sciences, Unit of Oncology and Molecular Pathology, University of Cagliari, Cagliari, Italy; 7 Department of Surgery, Colorectal Surgery Center, University of Cagliari, Cagliari, Italy; 8 Unit of Hereditary Cancer, IRCCS Ospedale Policlinico San Martino, Genoa, Italy; 9 Department of Oncology, University of Turin, Turin, Italy; 10 Candiolo Cancer Institute-FPO, IRCCS, Candiolo, Italy; 11 Biosciences Laboratory, Istituto Scientifico Romagnolo per lo Studio e la Cura dei Tumori (IRST) IRCCS, Meldola, Italy; 12 Department of Medical and Surgical Sciences for Children and Adults, Division of Medical Oncology, Policlinico di Modena Azienda Ospedaliero-Universitaria di Modena, Modena, Italy; 13 Department of Medical Oncology, University Hospital of Cagliari, Cagliari, Italy; 14 Medical Oncology Unit, Macerata General Hospital, ASUR Marche AV3, Macerata, Italy; 15 Unit of Hematology, A. Businco Oncology Hospital, Cagliari, Italy; 16 Department of Medical Sciences and Public Health, Occupational Health Unit, University of Cagliari, Cagliari, Italy; 17 Department of Biomedical Sciences, Cytomorphology Unit, University of Cagliari, Cagliari, Italy; 18 UCSF Weill Institute for Neurosciences, University of California San Francisco, San Francisco, CA USA; 19 Department of Neurology, University of California San Francisco, San Francisco, CA USA

**Keywords:** Clustered *PCDH*, Cancer methylation alteration, CpG islands, CTCF, Low grade glioma, LGG, Pilocytic astrocytoma, PA, Colorectal carcinoma, CRC, Colorectal adenoma, CRA, Gastric cancer, GC, Biliary tract cancer, BTC, Chronic lymphocytic leukemia, CLL

## Abstract

**Background:**

Clustered protocadherins (*PCDHs*) map in tandem at human chromosome 5q31 and comprise three multi-genes clusters: α-, β- and γ-*PCDH*. The expression of this cluster consists of a complex mechanism involving DNA hub formation through DNA-CCTC binding factor (CTCF) interaction. Methylation alterations can affect this interaction, leading to transcriptional dysregulation. In cancer, clustered *PCDH*s undergo a mechanism of long-range epigenetic silencing by hypermethylation.

**Results:**

In this study, we detected frequent methylation alterations at CpG islands associated to these clustered *PCDHs* in all the solid tumours analysed (colorectal, gastric and biliary tract cancers, pilocytic astrocytoma), but not hematologic neoplasms such as chronic lymphocytic leukemia. Importantly, several altered CpG islands were associated with CTCF binding sites. Interestingly, our analysis revealed a hypomethylation event in pilocytic astrocytoma, suggesting that in neuronal tissue, where *PCDH*s are highly expressed, these genes become hypomethylated in this type of cancer. On the other hand, in tissues where *PCDH*s are lowly expressed, these CpG islands are targeted by DNA methylation. In fact, *PCDH*-associated CpG islands resulted hypermethylated in gastrointestinal tumours.

**Conclusions:**

Our study highlighted a strong alteration of the clustered *PCDHs* methylation pattern in the analysed solid cancers and suggested these methylation aberrations in the CpG islands associated with *PCDH* genes as powerful diagnostic biomarkers.

**Electronic supplementary material:**

The online version of this article (10.1186/s13148-019-0695-0) contains supplementary material, which is available to authorized users.

## Background

Protocadherins (PCDHs) are type I transmembrane proteins containing 6 or 7 extracellular cadherin repeats, structurally similar to cadherins. They are characterized by a large molecular diversity, are broadly expressed and participate in cell-cell adhesion, predominantly in the nervous system establishing complex neural circuits [1].

*PCDHs* are classified as clustered and non-clustered protocadherins. The clustered *PCDH*s map in tandem at human chromosome 5q31 and comprise cluster α, cluster β and cluster γ genes (Human Genome Organization nomenclature *PCDHA@*, *PCDHB@* and *PCDHG@,* respectively), whereas the non-clustered *PCDH*s are distributed across the genome. A great variety of activities have been reported for clustered PCDHs. These molecules mediate homophilic interactions like most members of the cadherin superfamily [2]. The formation of these macromolecular complexes leads to the activation or inhibition of different signalling pathways through binding to the cytoplasmic domains of the PCDHs [3]. These transmembrane proteins regulate Wnt/β-catenin [4], PYK2 and FAK tyrosine kinases (involved in cell adhesion) [5, 6] and mTOR pathways [4], among others.

Wu and Maniatis first described the structure of protocadherin gene clusters [7, 8]. *PCDHA* and *PCDHG* gene clusters consist of variable exons that encode for the extracellular domain, the transmembrane domain and a short part of the cytoplasmic domain, and constant exons that encode for a shared C-terminal domain. In contrast, *PCDHB* gene cluster presents exons without a constant region. As *PCDHB* exons, each variable exon of *PCDHA* and *PCDHG* has its own promoter that is controlled by methylation [7, 8]. It has been reported that promoter stochastic choice, due to methylation changes and DNA-binding factor, and transcript splicing generate Pcdh diversity in neurons [9, 10]. This promoter choice and thus the transcription of clustered protocadherins depend on a complex mechanism where the CCTC binding factor (CTCF) plays an essential role. This zinc finger protein binds to a conserved sequence element (CSE) and a specific sequence element (SSE) located in the promoter, and to the enhancer element, a regulatory region downstream of each cluster, favouring genome looping [11, 12]. CTCF recognizes its DNA-binding sites, recruits the cohesion complex, whose members are Rad21, Smc1, Smc3 and SA2 [13], and allows the interaction of active promoters and specific enhancers through the formation of a hub [12]. Recent works also suggested that the binding of CTCF to the *Pcdha* cluster is regulated by transcription of a long non-coding RNA (lncRNA), initiated at a newly identified promoter within each *Pcdha* exon. Transcription of this antisense lncRNA mediates DNA demethylation of the CTCF binding sites, thus promoting CTCF binding [14]. Guo et al. found that forward-reverse orientation of the CTCF binding sites is also important for looping formation and enhancer-promoter interactions leading to cell-specific gene expression [15].

As mentioned before, *PCDH* expression is controlled by DNA methylation and its dysregulation is common in different types of cancer. In cancer pathogenesis, clustered *PCDH*s undergo a mechanism of long-range epigenetic silencing (LRES) by hypermethylation. Clustered *PCDH* gene silencing was found not only in tumour cell lines but also in different types of cancer including cervix, liver, lung, colon, breast and brain [4, 16–18]. Novak et al. detected hypermethylation and transcription downregulation in the three clustered *PCDH*s in breast cancer [17]. Other breast cancer studies showed that the abnormal DNA methylation of these gene families could be the consequence of the reduction of CTCF interaction with DNA due to CTCF aberrant expression or mutations in its binding domain [19, 20]. On the other hand, Guo et al. revealed that promoter methylation prevents or reduces CTCF binding to CSE [12]. Dallosso et al. also found hypermethylation of the majority of *PCDHA*, *PCDHB* and *PCDHG* in both adenomas and colorectal carcinomas, relative to normal tissue [4]. Moreover, these authors demonstrated that selected γ-PCDH are able to suppress Wnt activity in vitro [21]. In particular, PCDHGC3 negatively regulates Wnt and mTOR signalling. Interestingly, *PCDHGC3* has been found highly methylated only in carcinomas and not in previous stages and has been proposed as a driver for the progression from adenoma to carcinoma [4]. Thus, although the role of protocadherins in tumour development has not been fully established, it is suggestive that these proteins are involved in the regulation of key cellular pathways of cell death and proliferation. More recently, Liu et al. demonstrated that *PCDHGA7* downregulation is correlated with poor prognosis and *KRAS* genotypic status in colorectal cancer [22]. Waha et al. detected hypermethylation in *PCDHGA11* in astrocytoma, glioblastoma and glioma cell lines. Moreover, these authors found a significant correlation between *PCDHGA11* hypermethylation and downregulation in astrocytomas and glioma cell lines [16]. On the other hand, Kawaguchi et al. reported mosaic methylation and hypomethylation of the CpG islands (CGIs) associated with *Pcdha* cluster in mouse neuroblastoma cell lines [23]. Other *PCDHA*@ genes, *PCDHA4* and *PCDHA13*, have been found frequently hypermethylated in severe cervical neoplasia [18].

In the present study, we investigated the methylation status of clustered *PCDH*s in colorectal, gastric and biliary tract cancers (CRC, GC and BTC, respectively); pilocytic astrocytoma (PA); and chronic lymphocytic leukemia (CLL). Our results demonstrate that *PCDH*s frequently present alterations in their methylation status in solid cancers in contrast to blood cancer, suggesting the methylation alterations of these clustered genes as possible biomarkers for cancerogenesis.

## Methods

### Experimental discovery datasets

**Fig. 1:**
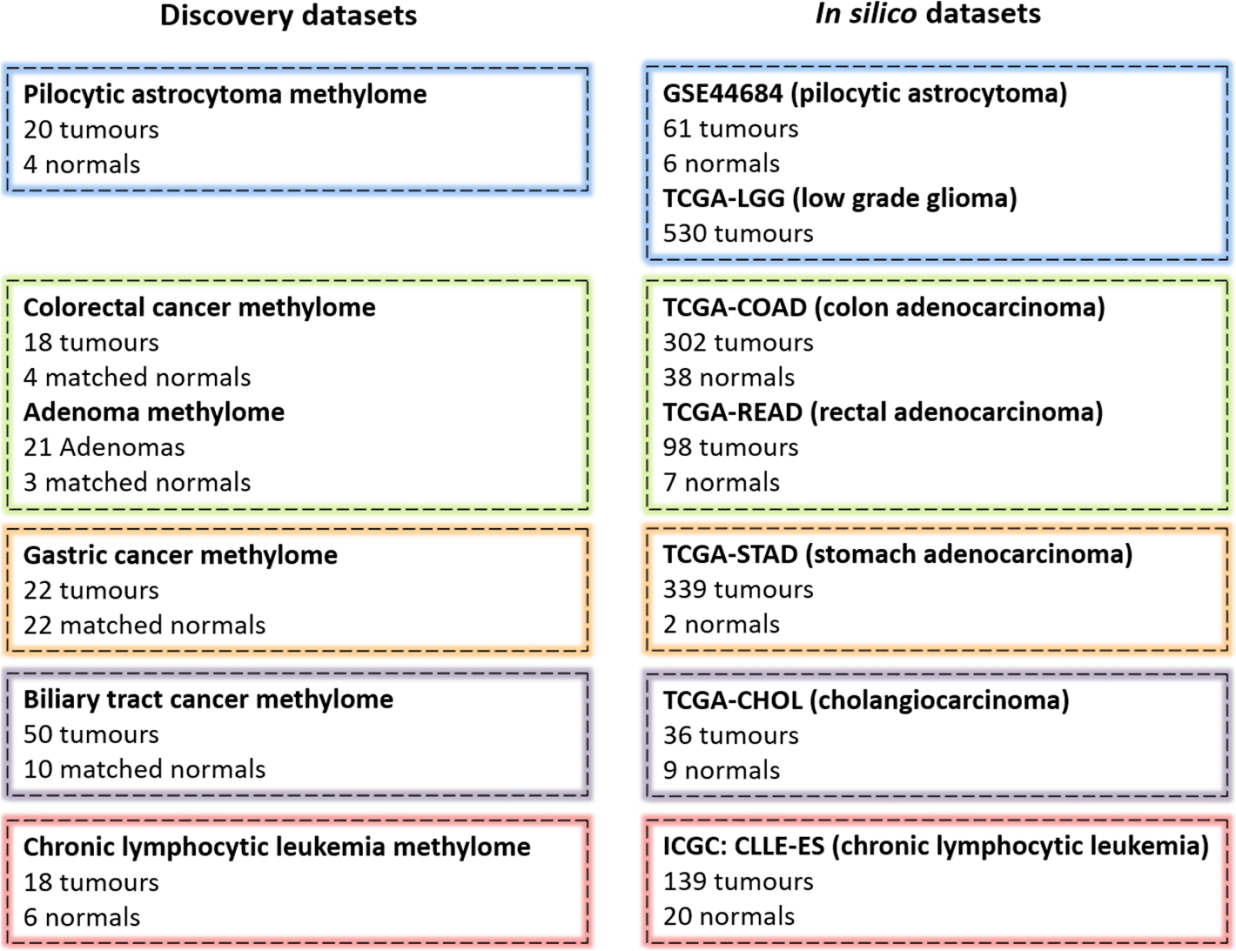
Description of sample sets used for the work. Discovery datasets: cancer samples collected for the study of methylome. In silico datasets: data used to validate the methylation alterations identified in clustered *PCDH*s, to explore the overall survival in relation to the identified aberrations and the correlation between methylation and expression of selected *PCDHG*s

Our experimental discovery dataset included DNA methylation data of four solid cancers (PA, CRC, GC and BTC) and one blood cancer (CLL) as summarized in Fig. 1. In particular, we analysed the following:

- 20 paediatric PAs, collected as part of the Italian National Program of Centralization of Paediatric Brain Tumour and four normal brain control samples obtained from temporal lobes of adult healthy individuals submitted to epilepsy routine examination;

- 18 primary CRC and four matched normal samples collected from the Department of General and Coloproctological Surgery, University of Cagliari (Italy); 21 colorectal adenomas (CRAs) and three matched normal intestinal mucosa controls obtained from the National Institute for Cancer Research of Genoa (Italy);

- 22 paired GC and normal samples collected from the Candiolo Cancer Institute-FPO, IRCCS, University of Turin (Italy);

- 50 BTCs whose 10 paired tumour and normal samples, obtained from the Department of Oncology, University of Cagliari (Italy) and the Scientific Institute Romagnolo for the Study and Treatment of Tumours (IRST) Srl – IRCCS, Meldola, FC (Italy);

- 18 CLLs and six normal blood control samples collected at the Haematology Department of the A. Businco Oncology Hospital, Cagliari (Italy).

### DNA extraction, bisulfite conversion and methylation assay

DNA was extracted from PA, CRC, CRA, GC and their respective normal fresh frozen tissues using DNeasy Blood & Tissue Kit (Qiagen).

DNA from BTC and matched normal formalin-fixed and paraffin-embedded (FFPE) samples was carried out by QIAamp DNA FFPE Tissue kit (Qiagen).

In the CLL study, DNA was isolated from peripheral whole blood lymphocytes using the DNA extraction 500 arrow® Kit (DiaSorin Ireland Ltd).

DNA quantity of all samples was analysed by spectrophotometric reading (NanoDrop) and by fluorometric reading (Quant-iT™ PicoGreen® dsDNA Assay Kit) and its quality was evaluated by electrophoresis in a 0.8% agarose gel.

All DNA samples were bisulfite converted using EZ DNA Methylation Gold Kit™ (Zymo Research).

In the BTC study, DNA extracted from FFPE samples underwent an additional quality control step using the Infinium FFPE QC kit (Illumina) prior bisulfite conversion. Subsequently, they were subjected to a restoration step using the Infinium HD FFPE Restore Kit (Illumina).

Genome-wide methylation analysis was performed by Illumina Infinium HumanMethylation27 BeadChips (27K) in PA study, Illumina Infinium HumanMethylation450 BeadChips (450K) in CRC and CLL studies and Illumina Infinium methylation EPIC BeadChips in GC and BTC studies. The number of probes mapping in *PCDHG*@ cluster in the different BeadChips are reported in Additional file 1: Figure S1. Further information and clinical data are available in Antonelli et al. (PA study) and Fadda et al. (CRC study) [24, 25].

### Methylation analyses

Illumina methylation 27K raw data were analysed as described in Antonelli et al. [24]. Differential methylation levels (Δβ) between PAs and normal brain samples were calculated by Illumina Custom model, as implemented in the Illumina GenomeStudio software. We selected only differentially methylated probes (Δβ values ≥ 0.2 or ≤ − 0.2, i.e. 20% differential methylation level) annotated in *PCDH* gene clusters with a *p* value threshold < 0.001. Hypermethylation was defined as Δβ values ≥ 0.2 and *p* value threshold < 0.001, while hypomethylation was defined as Δβ values ≤ 0.2 and *p* value threshold < 0.001

Illumina 450K and EPIC raw data were analysed using RnBeads as previously described [26, 27]. In brief, a differential methylation analysis between tumour and normal control samples was performed for each cancer type studied (CRC, CLL, GC and BTC). The normalization for the microarray signals was perfomed by Subset-quantile Within Array Normalization (SWAN) [28]. Corrected *p* values (Benjamini & Hochberg) were computed as previously described [26, 27]. In particular, combined *p* values were adjusted for the entire CpG sites on the arrays using false discovery rate (FDR). CpG loci were annotated according to Illumina Manifest to obtain a gene list based on HUGO Gene Nomenclature Committee (HGNC) database. We selected only *PCDH*-associated differentially methylated CGIs with Δβ values ≥ 0.2 or ≤ − 0.2 and an adjusted *p* value < 0.05. Hypermethylation was defined as Δβ values ≥ 0.2 and adjusted *p* value < 0.05, while hypomethylation was defined as Δβ values ≤ 0.2 and adjusted *p* value < 0.05. Since the results of this analysis were less robust in adenomas [25], we used the nominal threshold (*p* values < 0.05) in CRAs.

Finally, for CRC, CRA, GC and BTC, the mean methylation value of each altered CGI for each sample has been used in an analysis of UHC and visualized by Bioconductor package “ComplexHeatmap” [29].

Contingency table 2 × 1 was used to evaluate the statistical significance between methylation levels and microsatellite instability (MSI) status.

CGI annotations in tables and figures correspond to UCSC CGI names, indicating the number of CpG sites included in the CGI.

### CTCF binding site analysis

We explored whether the altered CGIs were associated with the CTCF binding sites. As mentioned before, both regions are included in the promoter [11]. CTCF binding sites’ genomic coordinates were obtained from ENCODE database [30]. CTCF binding sites and CGIs were considered as associated if their distance was lower than 1000 bp.

### Power calculation

The power of the methylation analyses was estimated based on the calculation of mean delta betas and standard deviations using data retrieved from the NCBI Gene Expression Omnibus (GEO) portal [31] under accession number GSE48684. Based on this preliminary data, we performed a two-sample *t* test power calculation obtaining that a statistical power of 0.8 would be guaranteed by analysing 30 samples in order to detect a differential methylation level of at least 10%, using a type I error of 10e− 8 (which takes into account the need to correct for multiple tests).

### In silico validation datasets

In silico methylation data from The Cancer Genome Atlas (TCGA), the NCBI GEO Portal and the International Cancer Genome Consortium (IGCG) Data Portal were used to validate the methylation alterations detected in the different cancer types analysed (Fig. 1). Methylation *β* values of the identified altered CGI were visualized using the web tool TCGA Wanderer [32, 33].

### In silico analyses

Additional in silico analyses were conducted using data from TCGA.

The database DNA Methylation and gene expression in Human Cancer (MethHC) [34] was used to compare the methylation status of selected *PCDH*s (*PCDHGC3*, *PCDHGC4*, *PCDHGC5*) in different types of cancer.

Xena Functional Genomics Explorer [35] allowed to perform a survival analysis and to study the correlation between methylation and expression of *PCDHG* C-type in LGG and between the altered N-shelf region or altered CGIs associated with gene promoters and the expression of these genes in TCGA-LGG, TCGA-COADREAD, TCGA-STAD and TCGA-CHOL.

## Results

### *PCDH* cluster: an aberrantly methylated region in solid cancer

Differential methylation analyses between cancer and their respective normal tissue samples were performed using experimental datasets and the results have been cross-validated in silico (Fig. 1). Differential methylation levels (Δβ) revealed that clustered *PCDH* were aberrantly methylated in all the solid cancers analysed. In fact, hypermethylation of CGIs associated with *PCDH* genes was among the most significant methylation alterations detected, even in BTC where methylation differences between tumour and normal samples were fewer and less pronounced than in the other cancers analysed. In CRC, the most altered CGI associated with *PCDH*G@ was the fouth most hypermethylated CGI and the fifth most significantly altered CGI among the 74 CGIs found aberrantly methylated in both CRC and CRA in our previous study [25]. The most altered *PCDHG*-associated CGIs in GC and BTC among the statistically significant hypermethylated CGIs (adjusted *p* value < 0.05) were ranked as 122/522 and 40/48, considering the Δβ, and 28/522 and 13/48, considering the *p* value, respectively. Interestingly, we detected a hypomethylation event in *PCDHG* cluster although we did not find any hypermethylated CGIs associated with *PCDH* in pilocytic astrocytoma. This region was 12/208 most hypomethylated in our discovery set. In contrast, we did not find any relevant methylation alterations in *PCDH*s in CLL. Overall, these data suggest that clustered *PCDH* methylation alterations are frequent events during tumorigenesis.

### *PCDH* alterations in pilocytic astrocytoma

**Fig. 2:**
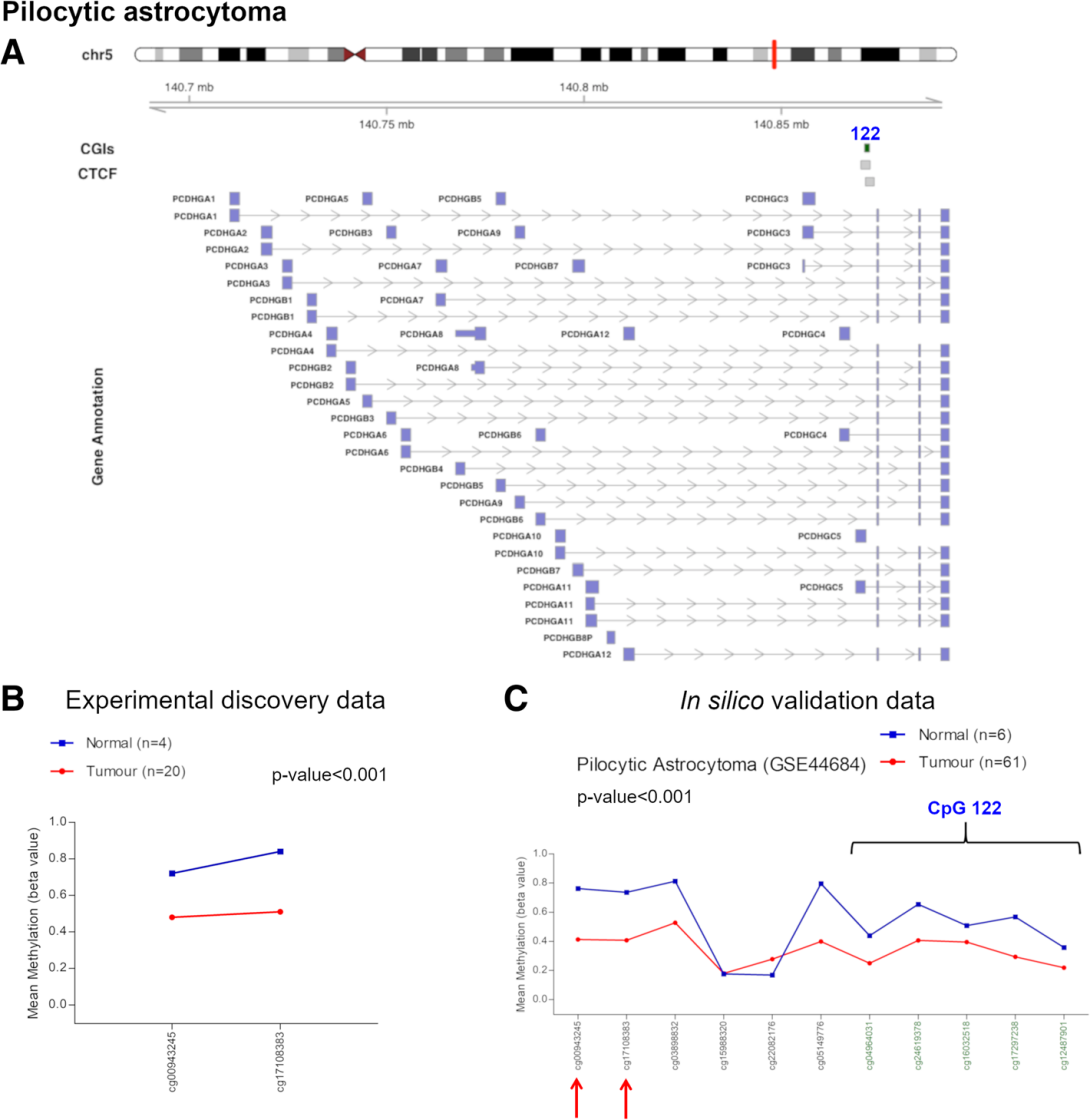
Methylation values obtained from the pilocytic astrocytoma discovery dataset and the in silico data. **a** Genomic organization of *PCDHG*@, including the localization of exons, CGIs (annotated with the UCSC CGI names) and CTCF binding sites. **b** Mean *β* values, resulting from the average of the samples (normal and tumour), of each probe obtained using Infinium HumanMethylation27 BeadChip. These two probes correspond to the N-shelf region of the CpG 122 (chr5:140871064-140872335), altered in our analysis. **c** Mean methylation values of each probe, belonging to the CpG 122 (green) and to its flanking region (black), obtained from the in silico dataset GSE44684. The red arrows indicate the two probes used in our experimental study

We evaluated the methylation status of *PCDH* cluster in 20 PA and four normal brain samples. We detected DNA hypomethylation (Δβ value = − 0.285) of a flanking region of a CGI (chr5:140871064-140872335, CpG 122) associated with the *PCDHG* cluster and two CTCF binding sites (Fig. 2a, b, Table 1) in PAs. The flanking region of this CGI is associated with *PCDHGC5* gene promoter (Fig. 2a, Table 1). This hypomethylation event was successfully cross-validated using in silico methylation data of pilocytic astrocytoma (GSE44684) (Fig. 2c). We could not investigate the methylation status of the CGI (chr5:140871064-140872335, CpG 122) since we did not have enough epigenome coverage using the Illumina Infinium HumanMethylation27 BeadChips, but in silico analysis revealed that also this CGI was hypomethylated in PA (Fig. 2c).

**Table 1:** Altered CGI flanking region in pilocytic astrocytoma

UCSC CGI	UCSC CGI name	CTCF binding site	Relation to UCSC CGI	Δβ value (PA-control)	Genes within region	Gene promoter-associated N-shelf
chr5:140871064-140872335	CpG 122	chr5:140870147-140872480	N-shelf	− 0.285	PCDHGA1, PCDHGA10, PCDHGA11, PCDHGA12, PCDHGA2, PCDHGA3, PCDHGA4, PCDHGA5, PCDHGA6, PCDHGA7, PCDHGA8, PCDHGA9, PCDHGB1, PCDHGB2, PCDHGB3, PCDHGB4, PCDHGB5, PCDHGB6, PCDHGB7, PCDHGC3, PCDHGC4, PCDHGC5	PCDHGC5
		chr5:140871308-140873492				

*Note*: *CGI* CpG island, *PA* pilocytic astrocytoma. CpG 122 corresponds to UCSC CGI name

### *PCDH* alterations in colorectal cancer

**Fig. 3:**
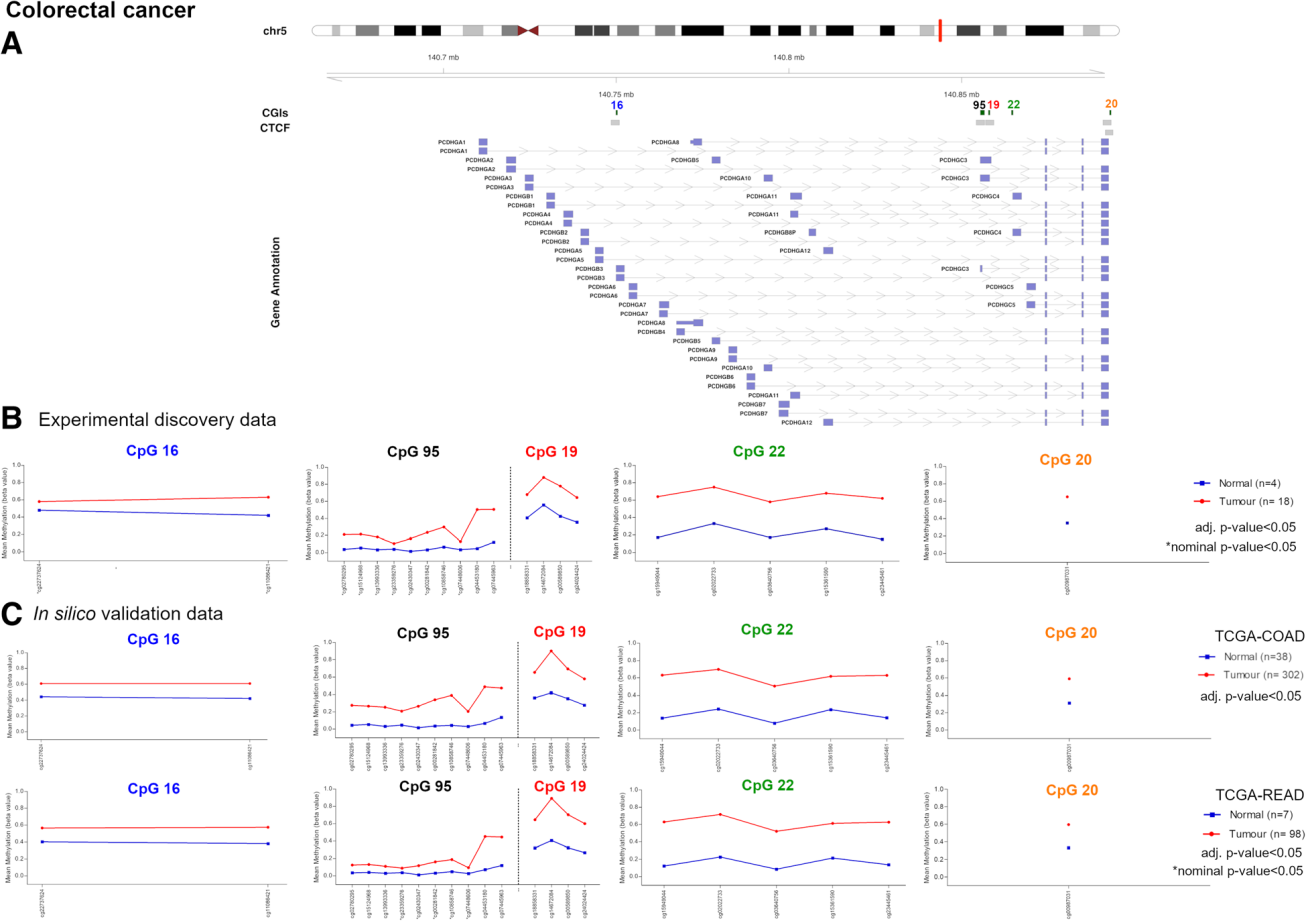
Methylation values obtained from the colorectal cancer discovery dataset and the in silico datasets. **a** Genomic organization of *PCDHG*@, including the localization of exons, CGIs (annotated with the UCSC CGI names) and CTCF binding sites. **b** Mean *β* values, resulting from the average of the samples (normal and tumour) of each probe of the altered CGIs obtained using Infinium HumanMethylation450 BeadChip. **c** Mean methylation values of each probe, belonging to the CpG 16, CpG 95, CpG 19, CpG 22 and CpG 20 (green), obtained from the in silico datasets TCGA-COAD and TCGA-READ

**Fig. 4:**
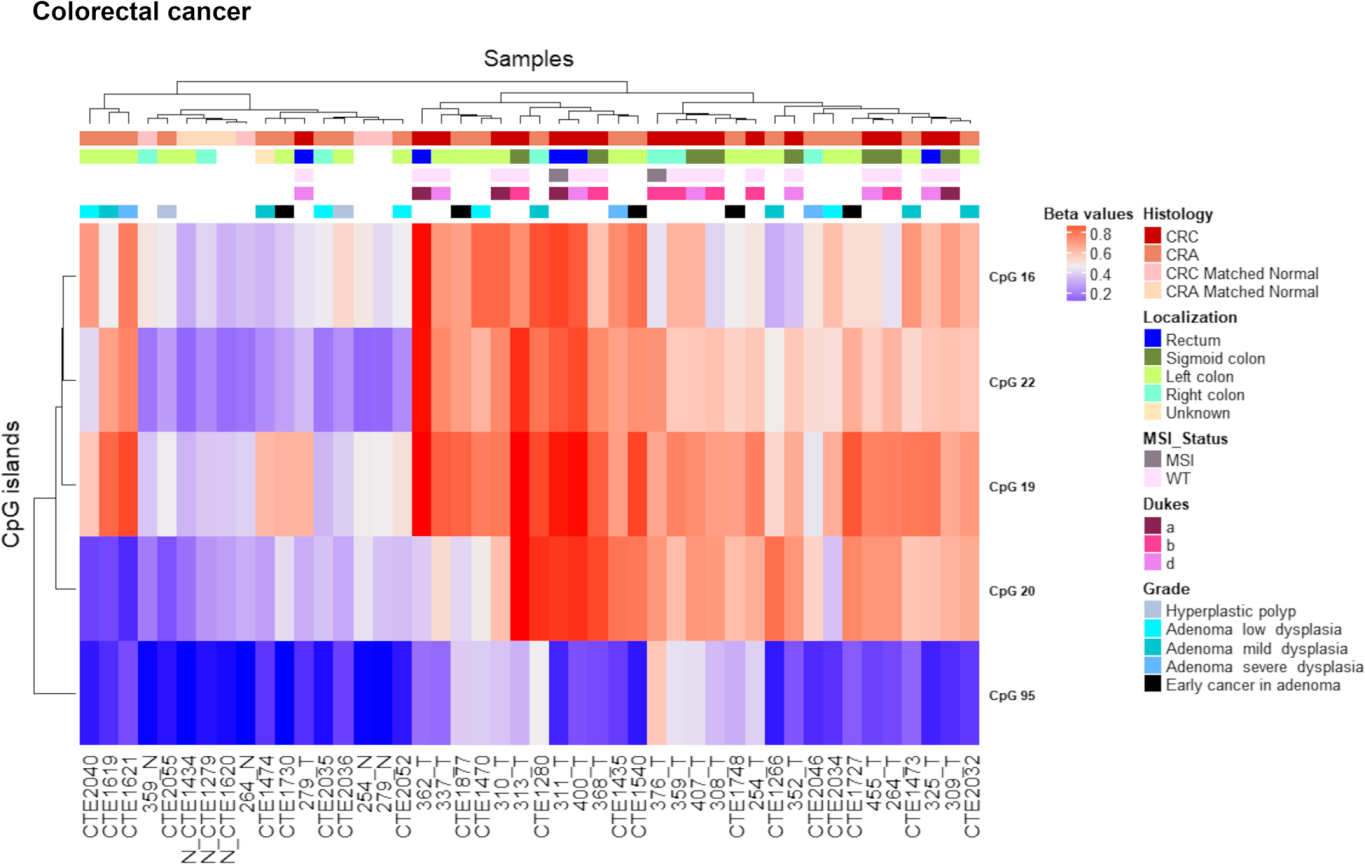
Colon discovery set unsupervised hierarchical clustering analysis based on the average methylation *β* value for each of the aberrantly methylated CGI. Heatmap obtained by UHC of CRC, CRA, CRC-matched normals and CRA-matched normals. All CRCs branched in a same group separated from control samples, except for sample 279T. Adenomas samples clustered randomly, 12 of them branched along CRCs and the others resembled the methylation status of normal samples. No correlation was observed between methylation profile and localization/subtype/staging in CRCs and CRAs. To the right of the heatmap, further information are reported: histology, localization, MSI status, Dukes and grade. CRC colorectal cancer, CRA colorectal adenoma, MSI microsatellite instability, WT wild-type

The differential methylation analysis conducted on 18 CRC and four normal samples revealed four significantly hypermethylated CGIs related to the *PCDHG* cluster (Fig. 3a, b, Table 2). All these altered CGIs, except one (chr5:140864527-140864748, CpG 22), were associated with CTCF binding sites (Fig. 3a, Table 2). To elucidate if these aberrations were early events in cancer process, we also performed a differential methylation analysis on 21 CRA and three control mucosae. This analysis revealed methylations alterations in the same CGIs altered in CRC (Fig. 3b, Table 2). Three altered CGIs mapped to promoter regions (Fig. 3a, Table 2). Of note, one of these CGIs (chr5: 140892913-140893189, CpG 20) was not associated with *PCDH*@ according to Illumina Manifest since it is located downstream the cluster and upstream *DIAPH1* gene. Nevertheless, we considered this altered CGI because it was significantly hypermethylated in both CRA and CRC. In general, the Δβ values were higher in carcinomas than in adenomas. On the contrary, one CGI (chr5:140750050-140750264, CpG 16) presented DNA methylation differences only in CRA samples (Table 2). The CGI located at chr5:140864527-140864748 (CpG 22) presented the highest differential methylation values in both tumour stages, i.e. Δβ value = 0.435 and 0.277, in CRC and CRA, respectively. Finally, beta values of the altered CGIs were visualized in a heatmap (Fig. 4). Unsupervised hierarchical clustering (UHC) showed a clear distinction between CRC and normal samples, except for 279T. In contrast, while 12 adenoma samples branched along with CRC samples, the methylation pattern of the other nine resembled that of normal samples. No association was observed between methylation values and clinical data (Fig. 4).

**Table 2:** Altered CGIs in colorectal cancer and colorectal adenoma

UCSC CGI	UCSC CGI name	CTCF binding site	Δβ value (CRC-control)	Δβ value (CRA-control)	Genes within region	Gene promoter-associated CGI
chr5:140750050-140750264	CpG 16	chr5:140748521-140750945	0.157*	0.200	PCDHGA1, PCDHGA2, PCDHGA3, PCDHGA4, PCDHGA5, PCDHGB1, PCDHGB2, PCDHGB3	PCDHGB3
chr5:140855386-140856620	CpG 95	chr5:140854218-140856648	0.200*	0.105	PCDHGA1, PCDHGA10, PCDHGA11, PCDHGA12, PCDHGA2, PCDHGA3, PCDHGA4, PCDHGA5, PCDHGA6, PCDHGA7, PCDHGA8, PCDHGA9, PCDHGB1, PCDHGB2, PCDHGB3, PCDHGB4, PCDHGB5, PCDHGB6, PCDHGB7, PCDHGC3	PCDHGC3
chr5:140857864-140858065	CpG 19	chr5:140856882-140859319	0.310	0.259	PCDHGA1, PCDHGA10, PCDHGA11, PCDHGA12, PCDHGA2, PCDHGA3, PCDHGA4, PCDHGA5, PCDHGA6, PCDHGA7, PCDHGA8, PCDHGA9, PCDHGB1, PCDHGB2, PCDHGB3, PCDHGB4, PCDHGB5, PCDHGB6, PCDHGB7, PCDHGC3	–
chr5:140864527-140864748	CpG 22	–	0.435	0.277	PCDHGA1, PCDHGA10, PCDHGA11, PCDHGA12, PCDHGA2, PCDHGA3, PCDHGA4, PCDHGA5, PCDHGA6, PCDHGA7, PCDHGA8, PCDHGA9, PCDHGB1, PCDHGB2, PCDHGB3, PCDHGB4, PCDHGB5, PCDHGB6, PCDHGB7, PCDHGC3, PCDHGC4	PCDHGC4
chr5:140892914-140893189	CpG 20	chr5:140890901-140893291	0.302	0.200	–	–
		chr5:140891594-140893806				

*Note*: *CGI* CpG island, *CRC* colorectal cancer, *CRA* colorectal adenoma. *Nominal threshold (*p* value < 0.05). CpG 16, 95, 19, 22 and 20 correspond to UCSC CGI name

We successfully validated all the aforementioned CGI alterations in data from TCGA (TCGA-COAD, TCGA-READ) (Fig. 3c). We observed that one CGI (chr5:140855386-140856620, CpG 95) was hypermethylated in COAD but not READ samples, with the exception of the most telomeric part of the CGI (cg04453180, cg07445963) (Fig. 3c). We observed the same methylation pattern in our discovery set. In general, the average CGI beta value was lower (0.125) in rectal cancer samples than in colon cancer samples (0.277). Moreover, this CGI presented lower methylation values in CRC than those observed in the other altered CGIs (Fig. 3, Fig. 4).

### *PCDH* alterations in gastric cancer

**Fig. 5:**
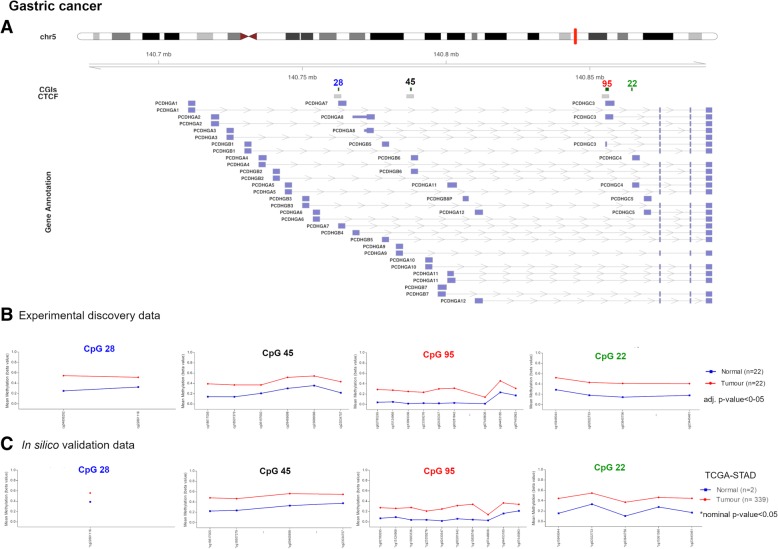
Methylation values obtained from the gastric cancer discovery dataset and the in silico dataset. **a** Genomic organization of *PCDHG*@, including the localization of exons, CGIs (annotated with the UCSC CGI name) and CTCF binding sites. **b** Mean *β* values, resulting from the average of the samples (normal and tumour), of each probe of the altered CGIs obtained using EPIC array. **c** Mean methylation values of each probe, belonging to the CpG 28, CpG 45, CpG 95 and CpG 22, obtained from the in silico datasets TCGA-STAD (450K array)

**Fig. 6:**
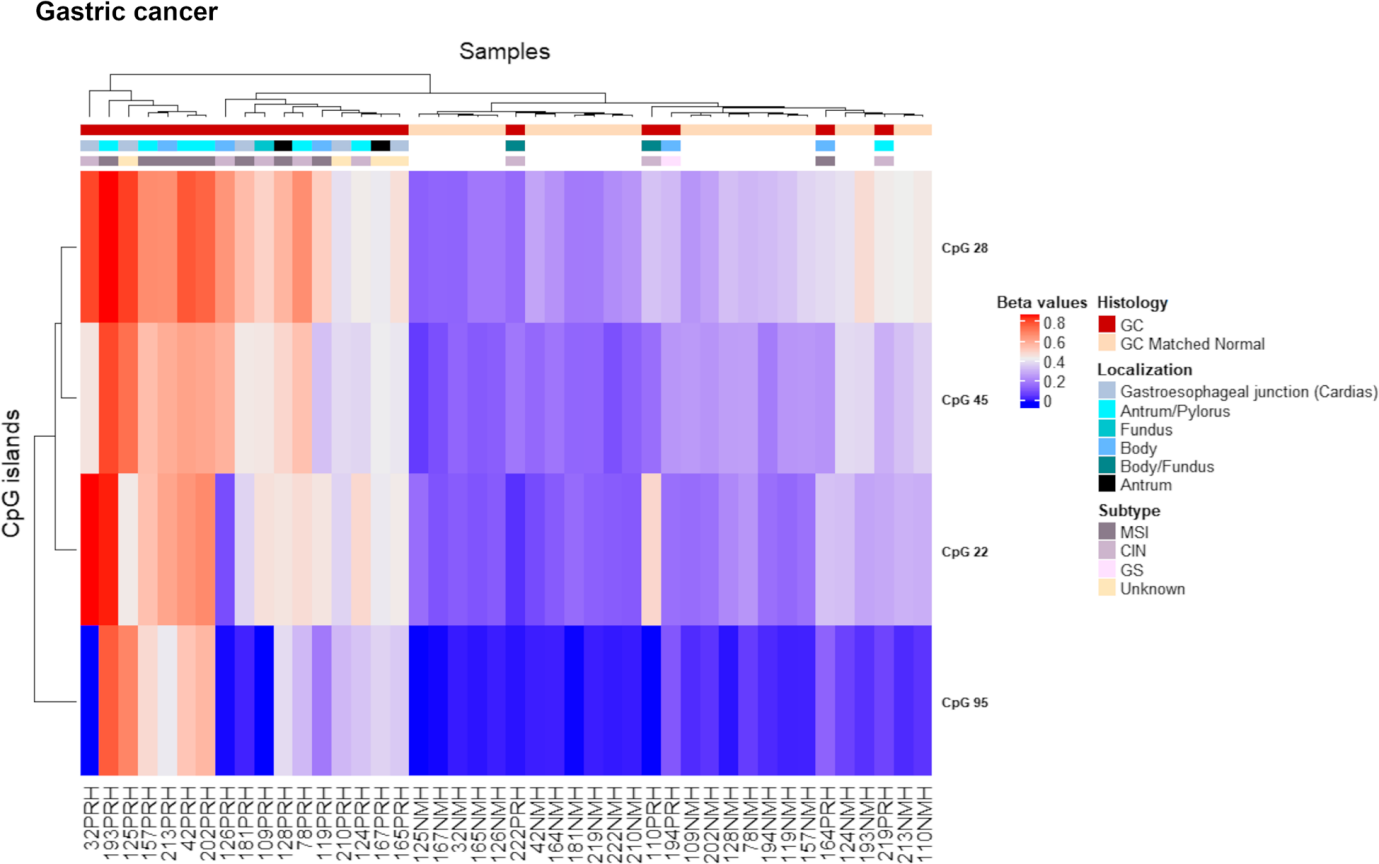
Gastric discovery set unsupervised hierarchical clustering analysis based on the average methylation *β* value for each of the aberrantly methylated CGI. Heatmap obtained by UHC of 22 gastric cancer samples and their matched normal samples. A group of GC with high methylation values branched together separated from normal samples and few GC samples that resembled the methylation pattern of controls. The UHC analysis also revealed another group of GC with a methylation profile between normal and tumour samples. To the right of the heatmap, further information are reported: histology, localization and subtype. GC gastric cancer, MSI microsatellite instability, CIN chromosomal instability, GS genomic stability

**Fig. 7:**
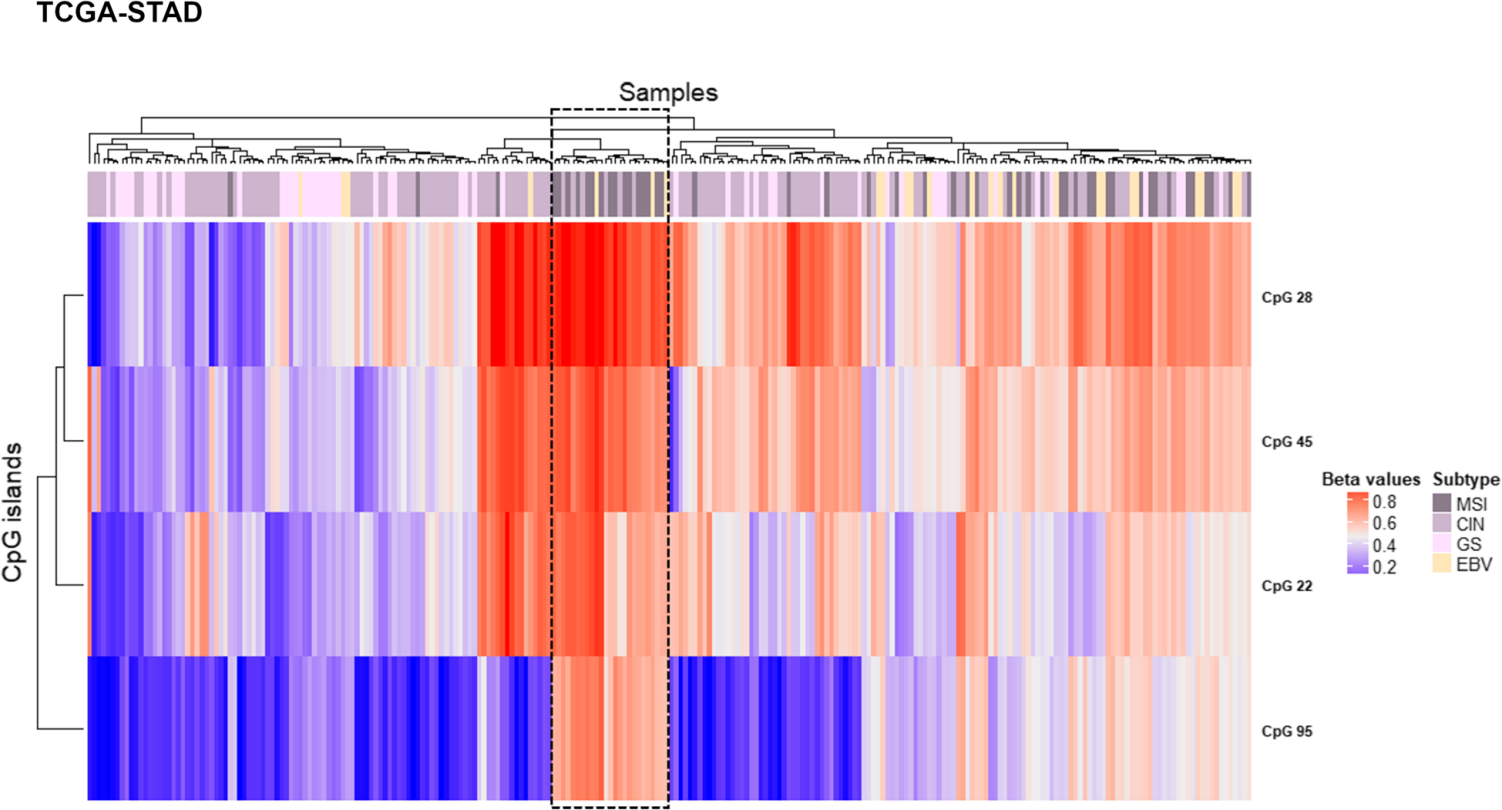
Gastric in silico set unsupervised hierarchical clustering analysis based on the average methylation *β* value for each of the aberrantly methylated CGI. Heatmap obtained by UHC of 248 gastric cancer samples. Two groups of GC branched separately according to their methylation levels. A subgroup with high methylation values in all CGIs is enclosed in a dashed box. To the right of the heatmap, subtype information are reported: MSI microsatellite instability, CIN chromosomal instability, GS genomic stability, EBV Epstein-Barr virus positivity

The differential methylation analysis between 22 gastric tumour and their matched normal samples revealed four significantly hypermethylated CGIs that were associated with CTCF binding sites, with the exception of CpG 22, and mapped to promoter regions (Fig. 5a, b, Table 3). These alterations were successfully cross-validated in silico using the TCGA stomach adenocarcinoma dataset (TCGA-STAD) (Fig. 5c). Of note, two of these CGI (CpG 22 and CpG 95) were also altered in CRCs. CpG 95 showed a similar methylation pattern as that observed in CRC, with low *β* values compared to the other altered CGIs (Fig. 5). UHC analysis allowed to distinguish a group of tumours (*N* = 7) characterized by high methylation values in all the altered CGIs, a group of tumours (*N* = 5) that branched along with normal samples and a third group of tumours (*N* = 10) whose alterations were intermediate between these two groups (Fig. 6). To note, eight out of nine MSI (microsatellite instability) samples were in the clusters of sole tumours and the remaining one clustered with the normal samples (Fig. 6). Thus, MSI was significantly more frequent in the group of tumours with high methylation values (*p* value = 2.0E− 02). To validate these results, we performed a UHC analysis using in silico TCGA-STAD methylation data for the four altered CGIs of samples with available molecular subtype categorization (*N* = 248). UHC revealed two clusters of tumours with different methylation levels (Fig. 7). In particular, 47 out of 49 MSI samples branched within the cluster displaying high methylation values, confirming that MSI-positive samples were strongly significantly more frequent in the group of tumours with high methylation values (*p* value = 1.3E− 10). Interestingly, the subgroup characterized by high *β* values in all CGIs (within the dashed box) mainly included MSI samples (16 out of 24). To note, 22 out of 25 patients with Epstein-Barr virus (EBV) infection clustered within the group of high methylation values (Fig. 7), implying that EBV infection was significantly more frequent in the group of highly methylated samples (*p* value = 1.4E− 04). Furthermore, paired samples with body/fundus localization presented lower mean Δβ values for each altered CGI than the selected threshold (CpG 28 = 0.119, CpG 45 = 0.106, CpG 95 = 0.067 and CpG 22 = 0.130). The in silico validation could not berelated to the location (because only two control samples were available).

**Table 3:** Altered CGIs in gastric cancer

UCSC CGI	UCSC CGI name	CTCF binding site	Δβ value (GC-control)	Genes within region	Gene promoter-associated CGI
chr5:140762401-140762768	CpG 28	chr5:140761029-140763470	0.241	PCDHGA1, PCDHGA2, PCDHGA3, PCDHGA4, PCDHGA5, PCDHGA6, PCDHGA7, PCDHGB1, PCDHGB2, PCDHGB3	PCDHGA7
chr5:140787447-140788044	CpG 45	chr5:140786247-140788714	0.210	PCDHGA1, PCDHGA2, PCDHGA3, PCDHGA4, PCDHGA5, PCDHGA6, PCDHGA7, PCDHGA8, PCDHGA9, PCDHGB1, PCDHGB2, PCDHGB3, PCDHGB4, PCDHGB5, PCDHGB6	PCDHGB6
chr5:140855386-140856620	CpG 95	chr5:140854218-140856648	0.212	PCDHGA1, PCDHGA10, PCDHGA11, PCDHGA12, PCDHGA2, PCDHGA3, PCDHGA4, PCDHGA5, PCDHGA6, PCDHGA7, PCDHGA8, PCDHGA9, PCDHGB1, PCDHGB2, PCDHGB3, PCDHGB4, PCDHGB5, PCDHGB6, PCDHGB7, PCDHGC3	PCDHGC3
chr5:140864527-140864748	CpG 22	–	0.243	PCDHGA1, PCDHGA10, PCDHGA11, PCDHGA12, PCDHGA2, PCDHGA3, PCDHGA4, PCDHGA5, PCDHGA6, PCDHGA7, PCDHGA8, PCDHGA9, PCDHGB1, PCDHGB2, PCDHGB3, PCDHGB4, PCDHGB5, PCDHGB6, PCDHGB7, PCDHGC3, PCDHGC4	PCDHGC4

*Note*: *CGI* CpG island, *GC* gastric cancer. CpG 28, 45, 95 and 22 correspond to UCSC CGI name

### *PCDH* alterations in biliary tract cancer

**Fig. 8:**
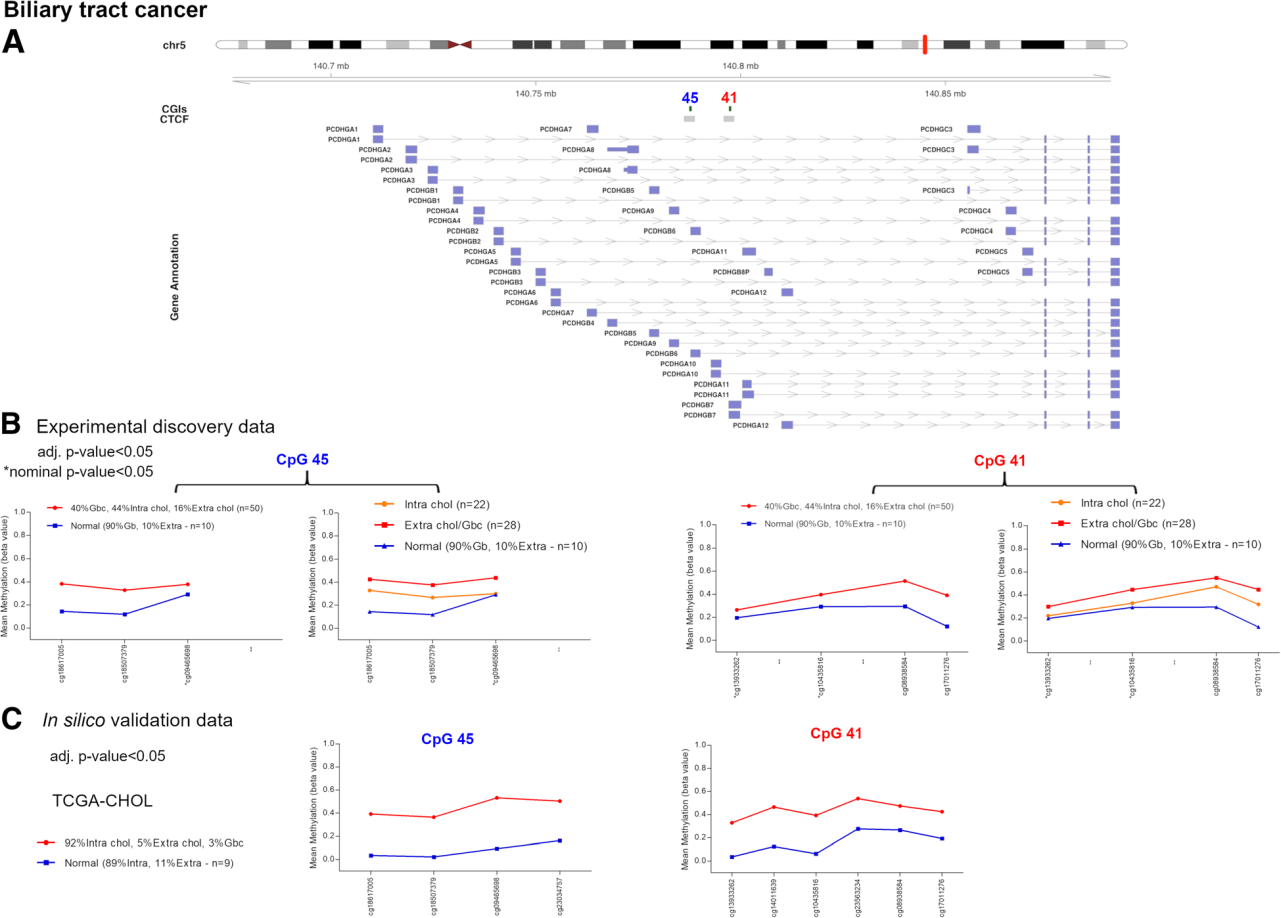
Methylation values obtained from the biliary tract cancer discovery dataset and the in silico dataset. **a** Genomic organization of *PCDHG*@, including the localization of exons, CGIs (annotated with the UCSC CGI name) and CTCF binding sites. **b** Mean *β* values, resulting from the average of the samples (normal and tumour) of each probe of the altered CGIs obtained using EPIC array. **c** Mean methylation values of each probe, belonging to the CpG 45 and CpG 41, obtained from the in silico datasets TCGA-CHOL (450K array). Tumour: Gbc, gallbladder cancer; Extra chol, extrahepatic cholangiocarcinoma; Intra chol, intrahepatic cholangiocarcinoma. Normal: Gb, gallbladder; Extra, extrahepatic; Intra, intrahepatic

The study conducted in BTC did not detect any CGI differentially methylated between BTC and matched normal samples according to our selection criteria. Nevertheless, two CGIs (chr5:140787447-140788044, CpG 45 and chr5:140797162-140797701, CpG 41), showed significant Δβ values with adjusted *p* values and were associated with two CTCF binding sites and promoter regions (Fig. 8a, b, Table 4). As previously mentioned, CpG 45 was altered in gastric cancer as well (Table 4).

**Table 4:** Altered CGIs in biliary tract cancer

UCSC CGI	UCSC CGI name	CTCF binding site	Δβ value (BTC-control)	Δβ value (Intrahepatic chol-control)	Δβ value (Extrahepatic chol-control)	Δβ value (Gallbladder cancer-control)	Δβ value (Extrahepatic/gallbladder-control)	Genes within region	Gene promoter-associated CGI
chr5:140787447-140788044	CpG 45	chr5:140786247-140788714	0.175	0.104	0.212	0.235	0.229	PCDHGA1, PCDHGA2, PCDHGA3, PCDHGA4, PCDHGA5, PCDHGA6, PCDHGA7, PCDHGA8, PCDHGA9, PCDHGB1, PCDHGB2, PCDHGB3, PCDHGB4, PCDHGB5, PCDHGB6	PCDHGB6
chr5:140797162-140797701	CpG 41	chr5:140795962-140798360	0.130	0.108	0.200	0.215	0.209	PCDHGA1, PCDHGA10, PCDHGA2, PCDHGA3, PCDHGA4, PCDHGA5, PCDHGA6, PCDHGA7, PCDHGA8, PCDHGA9, PCDHGB1, PCDHGB2, PCDHGB3, PCDHGB4, PCDHGB5, PCDHGB6, PCDHGB7	PCDHGB7

*Note*: *CGI* CpG island, *BTC* biliary tract cancer, *chol* cholangiocarcinoma. CpG 45 and 41 correspond to UCSC CGI name

BTC samples were heterogeneous and included 20 gallbladder carcinomas and 22 intrahepatic and eight extrahepatic cholangiocarcinomas. Hence, we analysed each group separately and found significant differences for these loci between intrahepatic cholangiocarcinomas and extrahepatic cholangiocarcinomas/gallbladder carcinomas (Fig. 8b, Table 4).

**Fig. 9:**
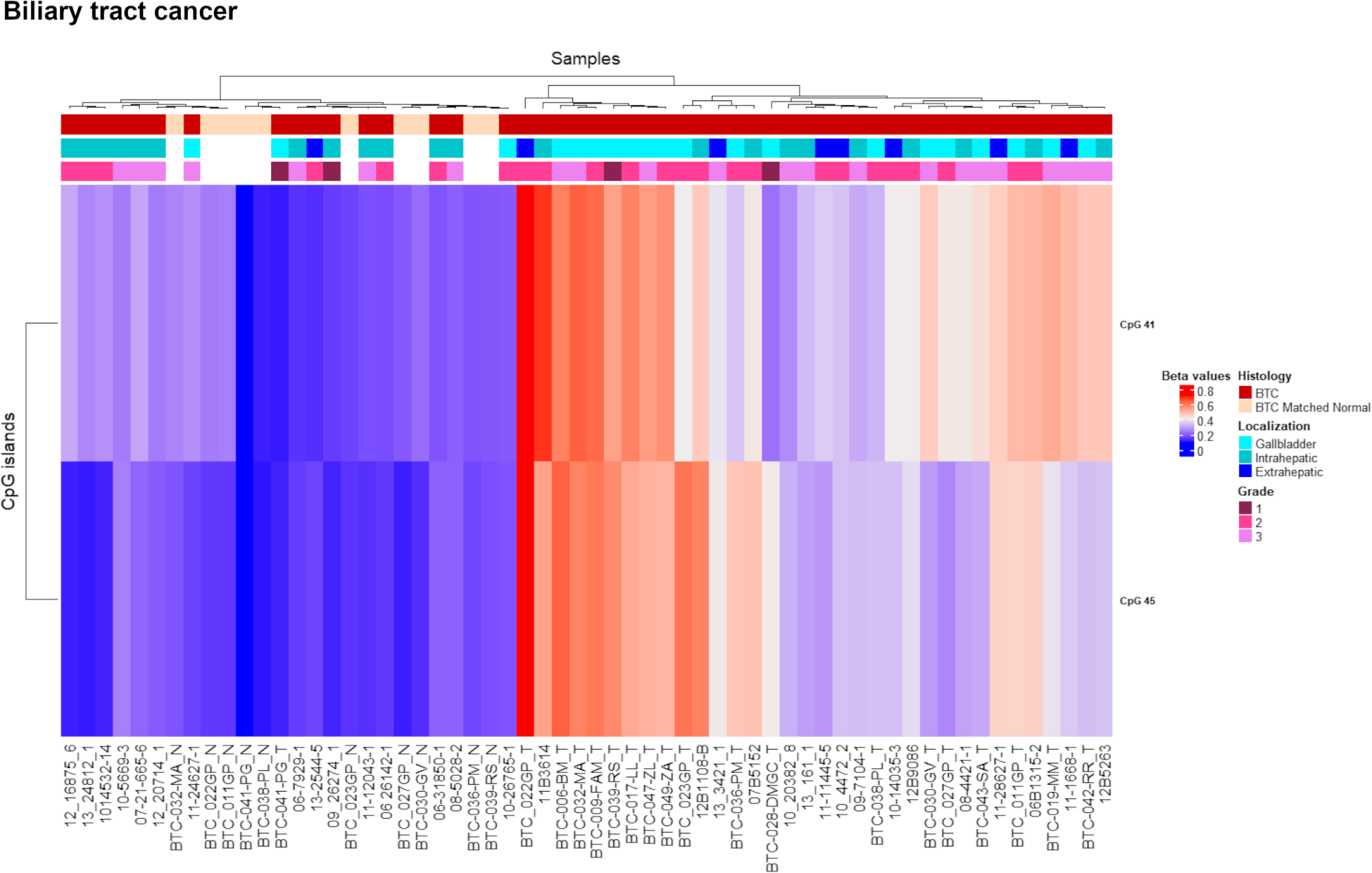
Discovery set unsupervised hierarchical clustering analysis based on the average methylation *β* value for the two aberrantly methylated CGIs. Heatmap obtained by UHC of 50 BTC samples and 10 matched normal samples. The UHC analysis clearly separated one group of sole tumours and another group including normal and tumoral samples. To the right of the heatmap further information are reported: histology, localization and grade. BTC, biliary tract cancer

UHC analysis showed a clear distinction between normal and the majority of tumoral samples (68%) and underlined the methylation differences among the three tumoral localizations (Fig. 9). In fact, the majority of the gallbladder (85%) and extrahepatic (87.5%) samples clustered together in the branch of sole tumours, while intrahepatic cholangiocarcinomas were distributed almost equally between the two main clusters.

The differential methylation of these CGIs was confirmed by the in silico methylation data (Δβ values = 0.370 and 0.278 for CpG 45 and CpG 41 respectively) (Fig. 8c) although these cases included 33 intrahepatic cholangiocarcinomas, two extrahepatic cholangiocarcinomas and one gallbladder cancer (TCGA-CHOL). To note, the normal samples of our discovery dataset included nine gallbladder and one extrahepatic tissues with average *β* values of 0.185 (CpG 45) and 0.227 (CpG 41), while in silico normal samples included eight intrahepatic and one extrahepatic tissues with average methylation values of 0.078 (CpG 45) and 0.160 (CpG 41).

### *PCDH* methylation pattern is not altered in chronic lymphocytic leukemia

Interestingly, these clustered genes behaved differently in a type of blood cancer, chronic lymphocytic leukemia, analysed by our group. Analysis of our experimental and in silico data (ICGC: CLLE-ES) did not reveal any significant methylation aberrations in *PCDH* clusters (Additional file 2: Table S1).

### Further in silico analyses

**Fig. 10:**
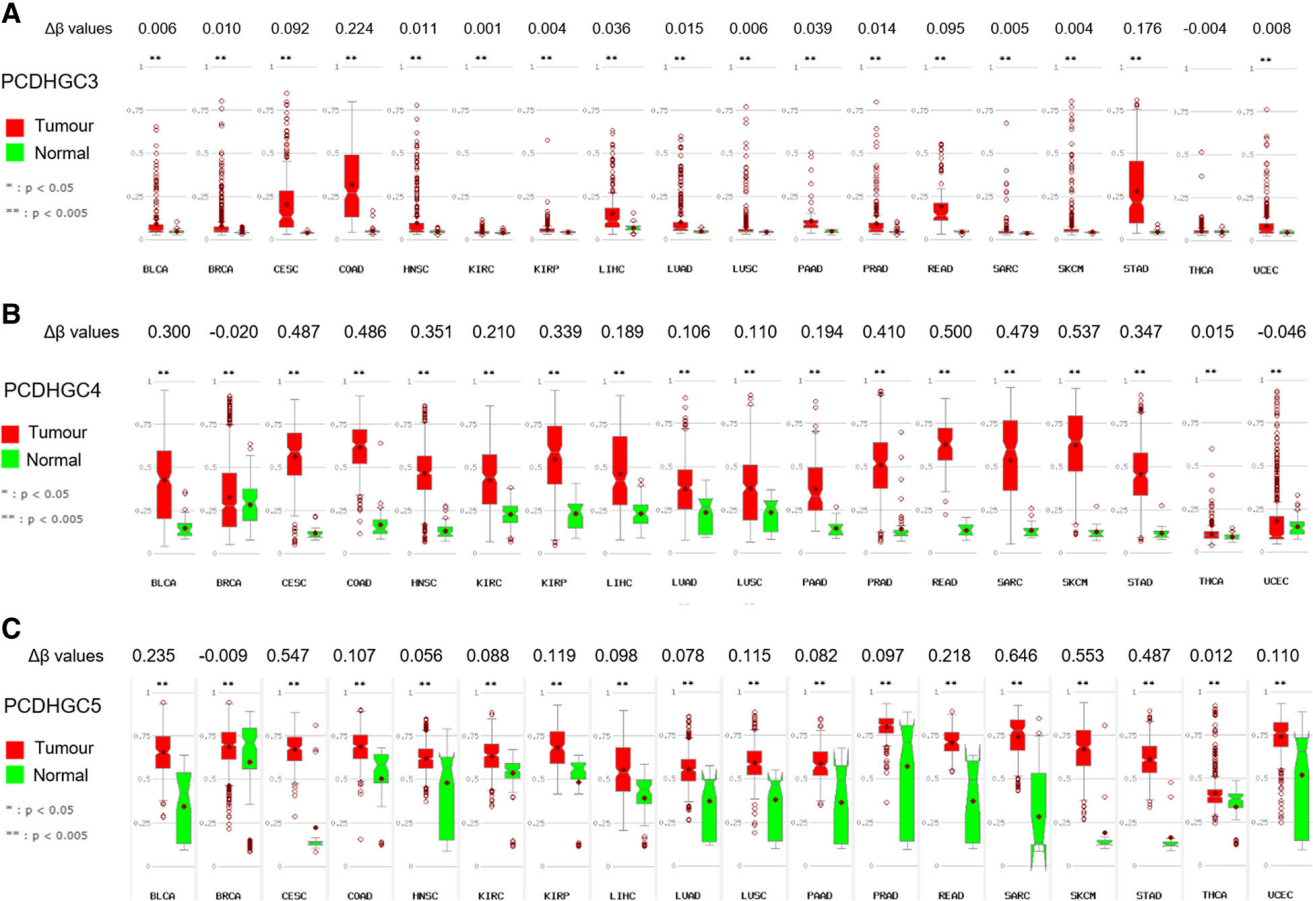
Box plots of the methylation values in tumour and normal tissues from different cancers, obtained from the in silico dataset TCGA. Differential methylation (Δβ) values of the CGIs of *PCDHGC3* (**a**), *PCDHGC4* (**b**) and *PCDHGC5* (**c**) were calculated between tumour and normal tissues. BLCA bladder urothelial carcinoma, BRCA breast invasive carcinoma, CESC cervical squamous cell carcinoma and endocervical adenocarcinoma, COAD colon adenocarcinoma, HNSC head and neck squamous cell carcinoma, KIRC kidney renal clear cell carcinoma, KIRP kidney renal papillary cell carcinoma, LIHC liver hepatocellular carcinoma, LUAD lung adenocarcinoma, LUSC lung squamous cell carcinoma, PAAD pancreatic adenocarcinoma, PRAD prostate adenocarcinoma, READ rectal adenocarcinoma, SARC sarcoma, SKCM skin cutaneous melanoma, STAD stomach adenocarcinoma, THCA thyroid carcinoma, UCEC uterine corpus endometrial carcinoma

To increase the robustness of our experimental results, we explored the methylation status of the altered CGIs associated with C-type *PCDHG* in different cancers, using the database MethHC (Fig. 10). As observed in Fig. 10a, *PCDHGC3* was significantly hypermethylated (Δβ value = 0.224) only in COAD. Differently, *PCDHGC4* and *PCDHGC5* were commonly hypermethylated in a large variety of tumours (Fig. 10b, c).

We tested whether the methylation alteration status of N-shelf region or CGIs annotated in promoter regions (Tables 1, 2, 3 and 4) could be associated with change in the expression pattern of the respective gene using TCGA-LGG, TCGA-COADREAD, TCGA-STAD and TCGA-CHOL data. We found a statistically significant negative correlation between methylation and gene expression (Additional file 3: Figure S2, Additional file 4: Figure S3, Additional file 5: Figure S4 and Additional file 6: Figure S5) except for CpG16 methylation and *PCDHGB3* gene expression in TCGA-COAD (Additional file 4: Figure S3).

**Fig. 11:**
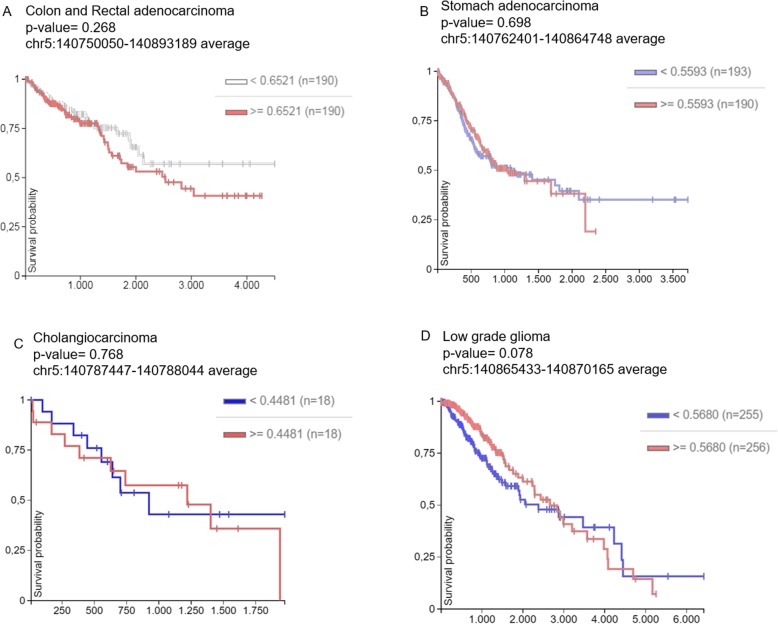
In silico survival curves of patients with colon and rectal adenocarcinoma (**a**), stomach adenocarcinoma (**b**), cholangiocarcinoma (**c**) and low grade glioma (**d**). The altered region detected in our research and used for this analysis is specified for each tumour type in the Kaplan-Meier plots (*x*-axis, survival time in days; y-axis, survival probability). Samples were divided into high and low methlyation value groups

**Fig. 12:**
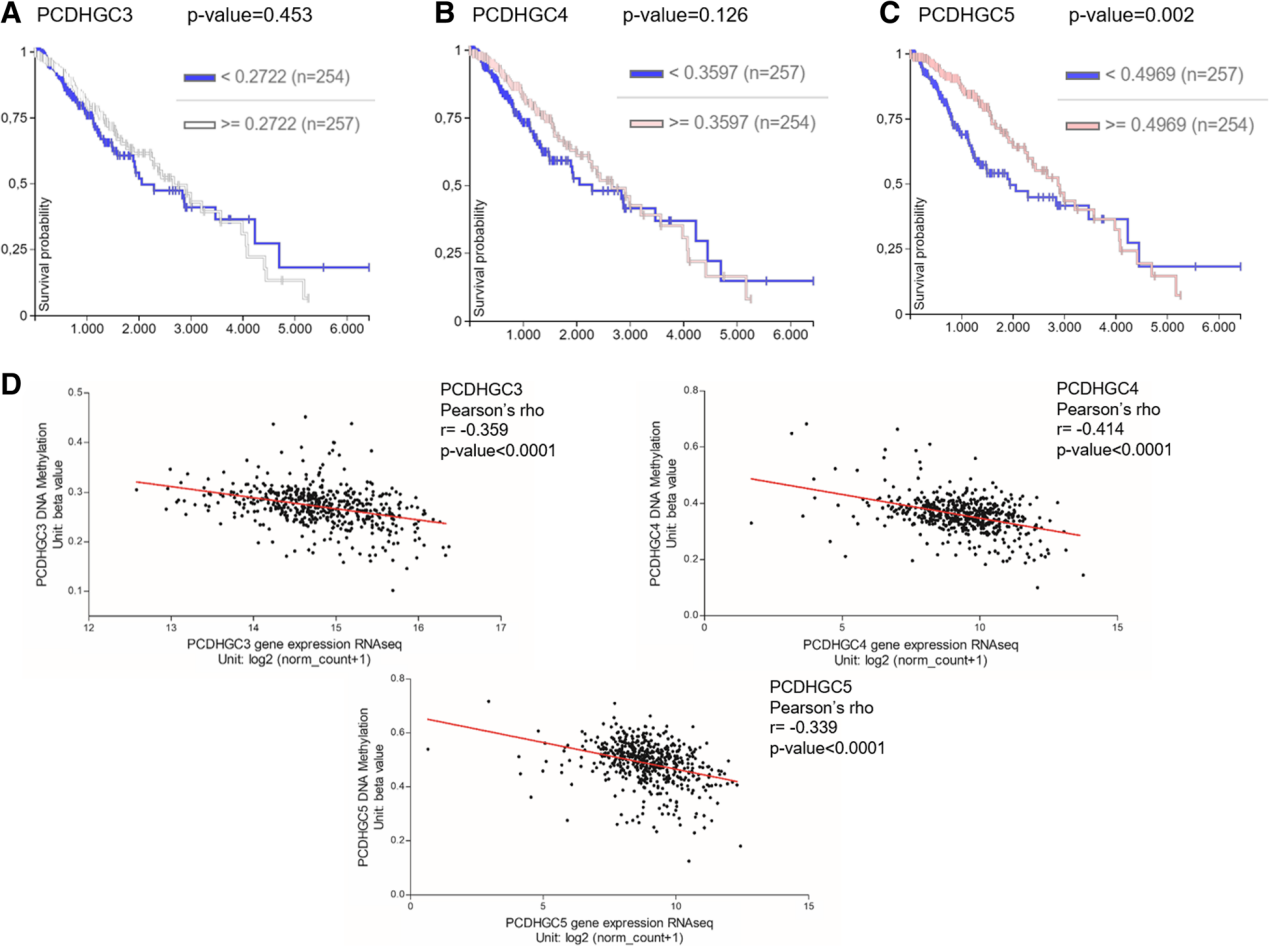
In silico analyses using the dataset TCGA-LGG. Survival curves of patients classified by high and low methylation *β* values of *PCDHGC3* (**a**), *PCDHGC4* (**b**) and *PCDHGC5* (**c**). **d** Correlation between methylation and expression level of each *PCDHG* C-type

We also investigated the correlation between the methylation status of the altered CGIs in the cancer types analysed in the current work and the overall survival using the web-tool UCSC Xena. Therefore, the survival curves were focused on the chromosome region, chr5:140750050-140893189 altered in CRC; chr5:140762401-140864748 in gastric cancer; chr5:140787447-140788044 in BTC; and chr5:140865433-140870165 in low grade glioma (LGG) (Fig. 11). Tumour samples were divided into high and low methylation *β* values groups. The Kaplan Meier plots showed a possible correlation only in LGG while the analyses did not reveal any significant differences between the two groups of patients with high and low methylation values in the other tumour types (Fig.11). In fact, in LGG, we observed an abrupt decrease of the survival probability in the first period of the survival time (*x*-axis) in patients with low *β* values (blue line) (Fig. 11d). Therefore, we focused the analysis in the C-types *PCDHGs* (Fig. 12). Firstly, the survival curves of each C-type isoform indicated that the low methylation values of *PCDHGC5* significantly correlate with a decrease of survival probability in the first period of this cancer type (Fig.12c). Secondly, the methylation level of each of the three isoforms, *PCDHGC3*, *PCDHGC4* and *PCDHGC5*, tended to negatively correlate with their expression levels, suggesting that aberrant methylation may be essential for their transcript regulation in LGG (Fig. 11d).

## Discussion

The epigenetic dysregulation of clustered *PCDH*s has been associated with brain disorders and with cancer as well [2]. In particular, the involvement of protocadherins in different types of tumours has been studied by several research groups [4, 16–18]. In the current experimental study, the CGIs related to *PCDH* clusters were markedly and significantly altered in the solid tumours analysed (Table 5). We detected, with a high statistical power, significant methylation alterations in CGIs associated with clustered *PCDHs* that were successfully cross-validated using a larger sample size from in silico TCGA datasets (Fig. 2, Fig. 3 and Fig. 5). Interestingly, all the detected altered regions, except CpG 19 and CpG 20, were associated with promoter regions. Since promoter choice is regulated by DNA methylation [36] and the variable region of each gene cluster contains several CpG sites [8], we analysed the correlation between methylation and expression of the altered CGIs mapping in promoter regions. Interestingly, a negative correlation between methylation and expression was detected (Additional files 3: Figure S2, Additional file 4: Figure S3, Additional file 5: Figure S4 and Additional file 6: Figure S5) except for CpG16 and *PCDHGB3* (Additional file 4: Figure S3). Specific members of the *PCDHG* cluster were also observed to be downregulated in CRC [4, 22]. Of note, despite the different methylation and expression aberrations within the clustered *PCDHs*, it should be taken into consideration the concept remarked by Han et al. and Chen and Maniatis that functional compensation is likely to occur among these gene clusters [37, 38].

**Table 5:** Summary of altered CGIs in the different cancer types

UCSC CGI	Relation to UCSC CGI	Methylation Analyses							
		CRA	CRC	GC	BTC			PA	CLL
					Intrahepatic chol	Extrahepatic chol	Gallbladder cancer		
chr5:140750050-140750264	Island	Hypermethylation	NA	NA	NA	NA	NA	ND	NA
chr5:140762401-140762768	Island	NA	NA	Hypermethylation	ND	ND	ND	ND	NA
chr5:140787447-140788044	Island	NA	NA	Hypermethylation	NA	Hypermethylation	Hypermethylation	ND	NA
chr5:140855386-140856620	Island	NA	Hypermethylation	Hypermethylation	NA	NA	NA	NA	NA
chr5:140857864-140858065	Island	Hypermethylation	Hypermethylation	NA	NA	NA	NA	ND	NA
chr5:140864527-140864748	Island	Hypermethylation	Hypermethylation	Hypermethylation	NA	NA	NA	NA	NA
chr5:140871064-140872335	N-shelf	NA	NA	NA	NA	NA	NA	Hypomethylation	NA
chr5:140892914-140893189	Island	Hypermethylation	Hypermethylation	NA	NA	NA	NA	ND	NA

*Note*: *CGI* CpG island, *CRA* colorectal adenoma, *CRC* colorectal cancer, *GC* gastric cancer, *BTC* biliary tract cancer, *chol* cholangiocarcinoma, *PA* pilocytic astrocytoma, *CLL* chronic lymphocytic leukemia, *NA* no alteration, *ND* not detected

In addition, differential methylation of C-type members of the *PCDHG* cluster was reported in silico in a great variety of cancers (Fig. 10). To explore whether the detected methylation alterations may also have an impact on tumour prognosis, we examined a possible association between high or low differential methylation values and the overall survival in silico (Fig. 11). The plots in Fig. 11 exhibited a trend in the correlation between patients with different levels of β values and the survival rate of LGG patients, while no association was found in the gastrointestinal tumour types. Furthermore, among the C-types *PCDHG* genes, *PCHDGC5* showed association with LGG survival probability (Fig. 12c) and could be a predictive biomarker. However, in this cancer, all C-type *PCDHGs* presented a significant negative correlation between methylation values and expression levels (Fig. 12d). In fact, as mentioned before, clustered *PCDH*s are mainly expressed in the nervous system while their expression is lower in other tissues [2, 39]. Thus, our results confirmed that hypermethylated genes in cancer are already lowly expressed in the respective normal tissues [25, 40, 41], while a tumour in a tissue with high expression, as in this case of gliomas, can undergo hypomethylation in this gene cluster. Finally, our experimental discovery data and the in silico analyses indicated that *PCDH* cluster genes undergo methylation pattern changes during gastrointestinal tumorigenesis.

The absence of significant methylation differences in clustered *PCDH*s genes in CLL suggested that they are not targeted by methylation during tumorigenesis in haematological neoplasms in contrast to solid tumours. An explanation for this result could be related to the cell adhesion function of *PCDH*s [1, 42] that is not essential in blood cancer for cell contact and tumour mass formation. Besides, our CLL analysis was also supported by the cross-validation in silico that revealed similar Δβ values (Additional file 2: Table S1). Further studies focused on other blood cancers are needed to support this finding.

The analysis of our gliomas data revealed a hypomethylation event (Δβ value = − 0.285) in the flanking region of a CGI associated with the *PCDHG* cluster, including the *PCDHG* C-type (Table 1). Although this hypomethylation did not involve the CGI itself but a flanking region, a previous work highlights the importance of alterations in this region in gene expression [43]. Supportive evidence was provided by the survival analysis considering only the flanking region of the CGI in LGG (Fig. 11d). This survival curve indicated that in the low-*β* values group of patients (blue line), the survival probability had an early reduction compared to the high-*β* values group (Fig. 11d). Thus, this hypomethylation event may have a prognostic implication in PA samples. Moreover, at the expression level, PCDHGs are essential during neuronal development and their knockdown or deficiency leads to loss of different neuronal cell types, synapse decrease or dendritic arborisation decline [37, 44, 45]. Therefore, the hypomethylation event could lead to the upregulation of this group of *PCDHG*s, suggesting that tumour cells need to behave as progenitor cells, i.e. returning to the conditions required during development. However, it should also be considered the possibility that the methylation status found in the tumour actually mirrors the cell of origin pattern clonally expanded [24, 40, 46–48]. In this case, it may not represent a cause or an effect of tumorigenesis, but still a cancer-specific clustered *PCDH* methylation pattern would remain a valuable biomarker. In addition, PCDHGs overexpression could be implicated in cell survival due to regulation of apoptotic signalling pathways [4] and interaction with cell-adhesion kinases [5, 49].

Our experimental data showed that the CGIs of clustered *PCDH*s in CRC are the most highly hypermethylated among the gastrointestinal tumours analysed (Tables 2, 3 and 4). UHC analysis revealed that all CRCs clustered together separated from normal samples, with the sole exception of sample 279T (Fig. 4), suggesting a strong methylation alteration of clustered *PCDH*s in CRC. Moreover, the hypermethylation of these CGIs could be early events during carcinogenesis because they are frequently found in our adenoma samples although some of them did not present methylation alterations. Of note, the Δβ value was always higher in CRCs than in adenomas, except for the CGI located at chr5:140750050-140750264 (CpG 16) (Table 2). As the values we are referring to, were average values, the differences observed between the two data sets could be due either to increased degree of methylation of each involved island in carcinomas compared to adenomas or to hypermethylation presence in more CRC samples than in adenomas. In this regard, we specifically looked at the *β* value for each sample for the selected islands. In fact, when we analysed adenomas, we found that while few of them branched nearby normal mucosa samples (CTE1279, CTE1434 and CTE1620), the remaining ones grouped on separated branches and some of them more closely resembled the methylation pattern of carcinomas (Fig. 4). To complement the analysis, we did not find any correlation between *PCDH*s methylation alterations and the grade of carcinogenesis in adenoma. In fact, as we observed in Fig. 4, the adenomas clustered randomly according to the disease grade.

Interestingly, our experimental methylation studies showed that some CGI alterations were common in different cancers (gastric, biliary tract and colorectal cancer) and others were specific for each cancer-type but they were all associated with the *PCDHG* cluster (Table 5). Interestingly, CpG 22, the most hypermethylated CGI in CRC was also the most hypermethylated in GC. Since this CGI was also hypermethylated in CRA indicating that it is an early event in CRC tumorigenic, it is likely that this event can occur early also during GC tumorigenesis. Other studies have previously found methylation alterations of *PCDHG* cluster in gastrointestinal tumours, including colon cancer [4]. We did not detect significant hypermethylation in the other two *PCDH* clusters in contrast to Dallosso et al. These events could be related to the wide expression of PCDHG cluster in embryonic and adult tissues, while PCDHA cluster is specifically expressed in the nervous system [4].

Furthermore, the detected methylation aberrations seem to be frequent events in gastrointestinal tumours, some involved in tissue-specific mechanisms and others in common mechanisms. In particular, we found a difference in the methylation pattern of CpG 95 between rectal and colon cancer samples suggesting that the identified alteration may be specific of colon localization. From a clinical point of view, this may be important because it could provide broad-spectrum and tissue-specific tumour biomarkers. Similar differences among localizations have been detected in GC and BTC samples. In fact, GC methylation analysis revealed that Δβ values of all the four altered CGIs did not reach our differential methylation threshold in paired samples localized in body/fundus. BTC results showed differences in Δβ values of two CGIs between localizations suggesting that the detected methylation alterations might reach higher *β* values in gallbladder/extrahepatic. It is important to mention that normal samples used in the current study were localized in gallbladder/extrahepatic ducts. Therefore, we cannot exclude that we did not observe any alterations in intrahepatic tumours because of the lack of their matched normal tissue samples. In fact, in silico data, where most of tumoral and normal samples had an intrahepatic localization, revealed marked methylation alterations of both CGIs. Thus, future analyses should be performed comparing tumoral samples to their coupled normal localization.

We investigated whether other clinical characteristics were associated with methylation alterations in the different cancers analysed, finding an association between hypermethylation and MSI status only in GC as reported by other authors [50, 51]. In fact, tumour samples with MSI branched together except for 164PRH sample that clustered along normal samples (Fig. 6). We successfully validated these results using in silico TCGA-STAD methylation data (Fig. 7). Moreover, in silico EBV-positive samples displayed high methylation levels for the altered CGIs. This result agrees with previous evidence reported in TCGA-STAD cohort where EBV-positive samples presented extreme CpG island methylator phenotype (CIMP) [52].

The association between molecular subtypes and methylation values could be also observed in the other gastrointestinal tumours. Due to the lack of these molecular data for both our experimental and in silico cohort, we could not evaluate a possible association in BTC. Given that our CRC samples clustered together in a group with high values for all the altered CGI and included two MSI samples, no association between methylation and MSI status can be detected. Furthermore, our previous analysis of TCGA-COAD and READ methylation data for 74 CGIs, including two *PCDH*-associated CGIs (CpG 19 and CpG 22), revealed that most CRC samples clustered in the group of tumours displayed high *β* values [25], confirming that high methylation levels of the analysed CGI alterations are not related to different molecular status.

Importantly, we included in our methylation analyses the position of the CTCF binding sites, possibly associated with the CGIs. As mentioned before, these sites are most likely related to the clustered *PCDHs* transcription through the formation of DNA loops mediated by CTCF interactions [11, 15]. Since methylation regulates CTCF binding [12], the methylation abnormalities detected in our experimental results could avoid or modify the hub formation by blocking the interaction between the CTCF protein and the neighbouring binding sites, consequently regulating *PCDHG* cluster transcription. Previous functional studies [12, 14, 20] have already shown that DNA methylation aberrations are associated to alteration of CTCF binding to DNA.

This study, although suggesting the evaluation of the clustered *PCDH*-associated CGIs methylation levels as a tumour biomarker in different types of cancer, has some limitations that can be overcome by more detailed future studies. A technical limitation is certainly due to the use of different types of arrays in the different cases, in particular, the 27K array for PAs, which therefore does not allow us to draw more definitive conclusions in the opposite methylation patterns observed in pilocytic astrocytomas compared to gastrointestinal tumours. In fact, further studies analysing DNA methylation alterations associated to *PCDH* cluster genes in additional brain tumours are needed to confirm the correlation between hypomethylation in cancer and normal tissue expression. Furthermore, an aspect that is certainly worth investigating is the lack of an experimental expression analysis and further functional analyses aimed to understand if and how the identified methylation alterations play a role in the tumorigenesis of the different tumours analysed.

## Conclusions

Although several authors have conducted analyses in clustered *PCDH*s, this work highlighted that methylation alterations of *PCDHG*@ are among the most statistically significant aberrations in solid cancers. Moreover, our results suggest that in neuronal tissue, where *PCDH*s are highly expressed, this gene cluster becomes hypomethylated in pilocytic astrocytomas, while in tissues where *PCDH*s are lowly expressed, this cluster is targeted by DNA methylation. These epigenetic aberrations in the CGIs associated to *PCDHG*@ genes could be useful to consider specific members of this cluster as possible biomarkers. Nevertheless, further research is necessary to elucidate their function and their expression regulation in each tumour type.

## Additional files

Additional file 1: **Figure S1.** BeadChips cPCDHG probes. (TIFF 1308 kb)

Additional file 2: **Table S1.** CGIs methylation values in CLL. (DOCX 13 kb)

Additional file 3: **Figure S2.** In silico correlation analysis between methylation and expression level of *PCDHGC5* in TCGA-LGG. Correlation analysis between the altered N-shef associated CGI, localized in the promoter region of *PCDHGC5*, using the TCGA-LGG dataset. CpG 122 corresponds to UCSC CGI names. (TIFF 500 kb)

Additional file 4: **Figure S3.** In silico correlation analysis between methylation and expression level of specific *PCDHGs* in TCGA-COADREAD. Correlation analysis between the altered CGIs, localized in the promoter region of *PCDHGB3*, *PCDHGC3* and *PCDHGC4*, using the TCGA-COADREAD dataset. CpG 16, 95 and 22 correspond to UCSC CGI names. (TIFF 940 kb)

Additional file 5: **Figure S4.** In silico correlation analysis between methylation and expression level of specific *PCDHGs* in TCGA-STAD. Correlation analysis between the altered CGIs, localized in the promoter region of *PCDHGA7*, *PCDHGB6*, *PCDHGC3* and *PCDHGC4*, using the TCGA-STAD dataset. CpG 28, 45, 95 and 22 correspond to UCSC CGI names. (TIFF 1196 kb)

Additional file 6: **Figure S5.** In silico correlation analysis between methylation and expression level of specific *PCDHGs* in TCGA-CHOL. Correlation analysis between the altered CGIs, localized in the promoter region of *PCDHGB6* and *PCDHGB7*, using the TCGA-CHOL dataset. CpG 45 and 41 correspond to UCSC CGI names. (TIFF 557 kb)

## Data Availability

The datasets analysed during this study are available from the corresponding author on reasonable request.
