# Supplementary figures and images for: Clustered protocadherins methylation alterations in cancer
Source: Clin Epigenetics. 2019 Jul 9;11:100. doi: 10.1186/s13148-019-0695-0 (PMC6617643; doi:10.1186/s13148-019-0695-0)

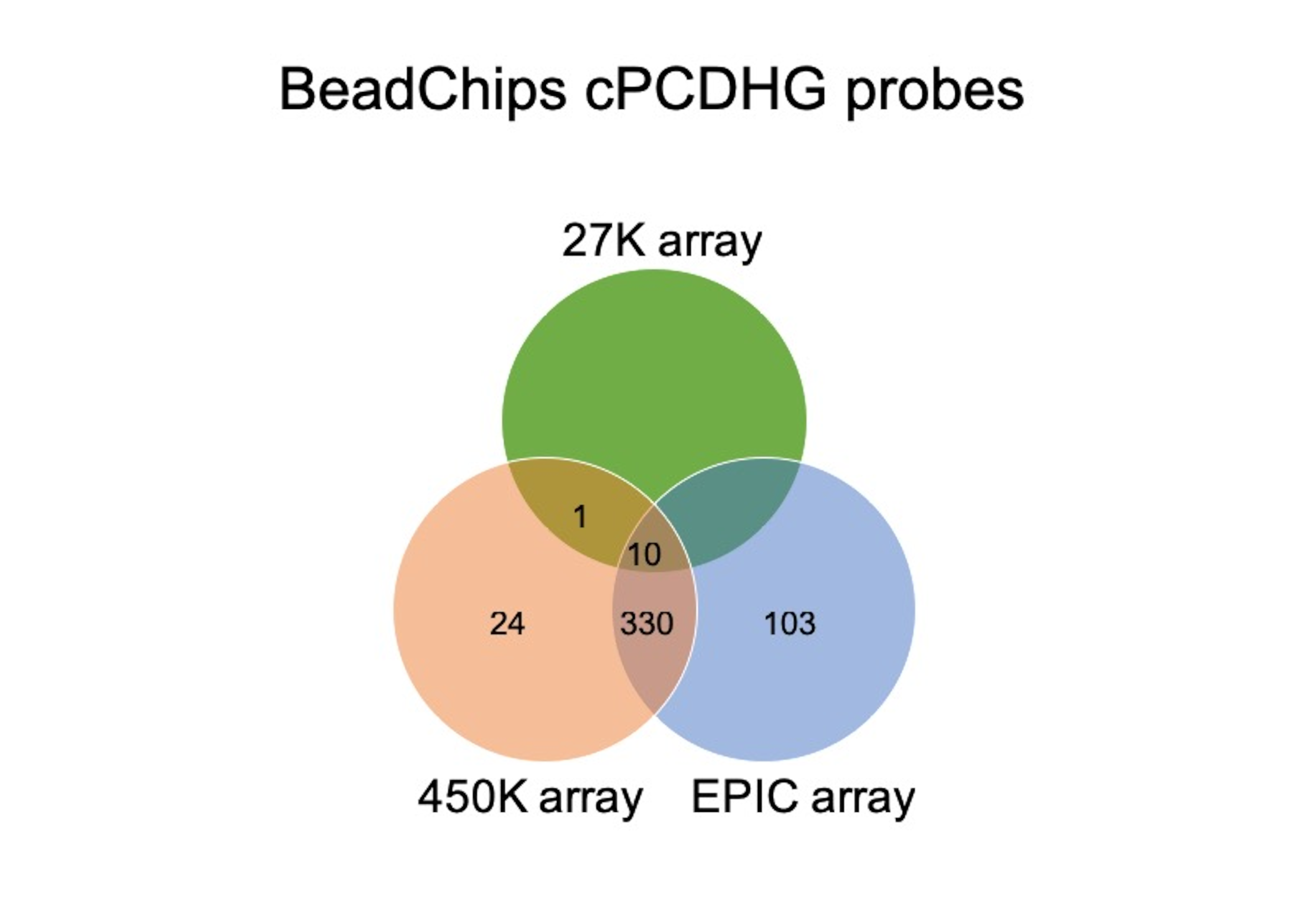

Supplement: Supplementary file 1 — Figure S1. BeadChips cPCDHG probes. (TIFF 1308 kb) [file 13148_2019_695_MOESM1_ESM.tiff]

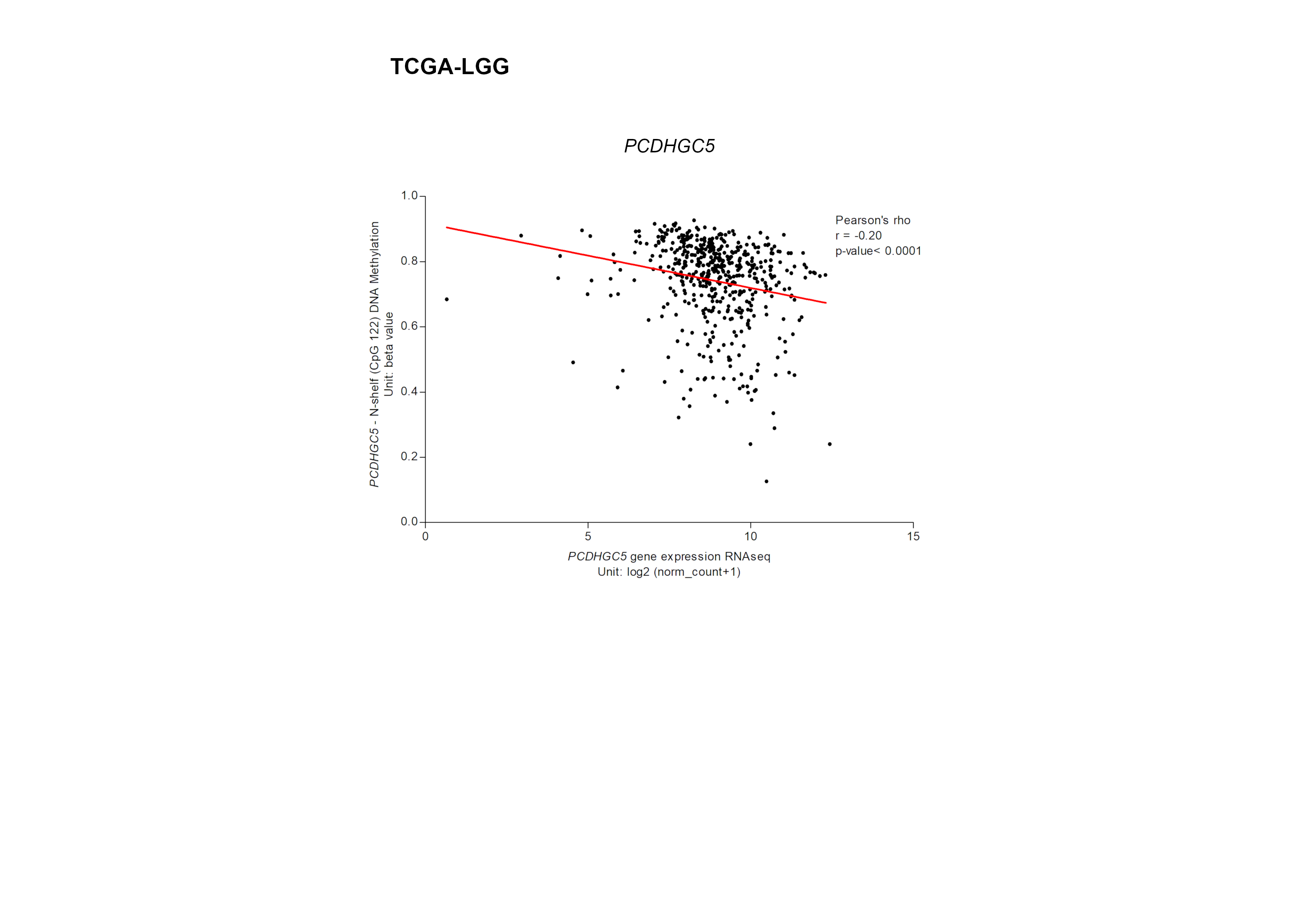

Supplement: Supplementary file 3 — Figure S2. In silico correlation analysis between methylation and expression level of PCDHGC5 in TCGA-LGG. Correlation analysis between the altered N-shef associated CGI, localized in the promoter region of PCDHGC5, using the TCGA-LGG dataset. CpG 122 corresponds to UCSC CGI names. (TIFF 500 kb) [file 13148_2019_695_MOESM3_ESM.tiff]

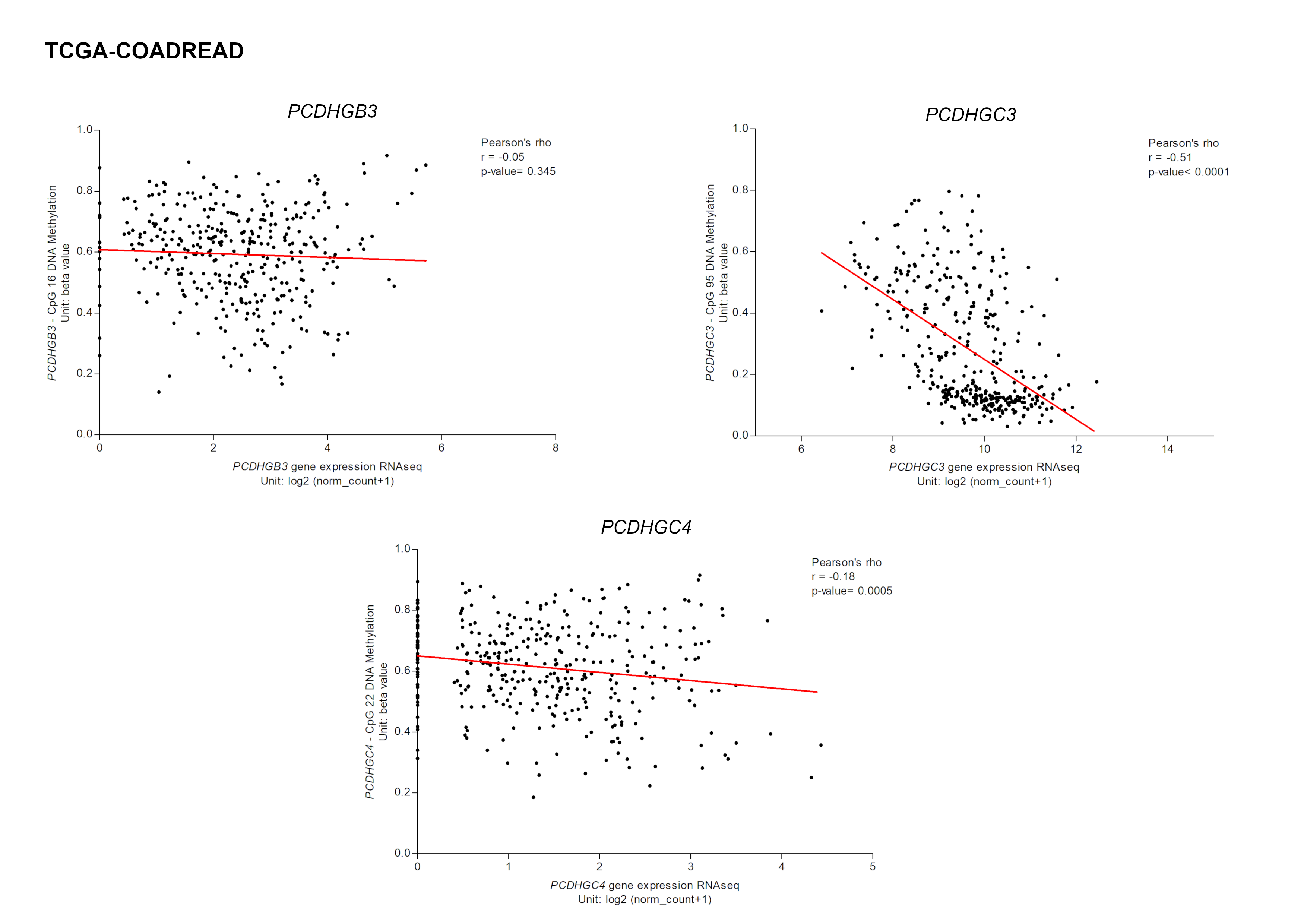

Supplement: Supplementary file 4 — Figure S3. In silico correlation analysis between methylation and expression level of specific PCDHGs in TCGA-COADREAD. Correlation analysis between the altered CGIs, localized in the promoter region of PCDHGB3, PCDHGC3 and PCDHGC4, using the TCGA-COADREAD dataset. CpG 16, 95 and 22 correspond to UCSC CGI names. (TIFF 940 kb) [file 13148_2019_695_MOESM4_ESM.tiff]

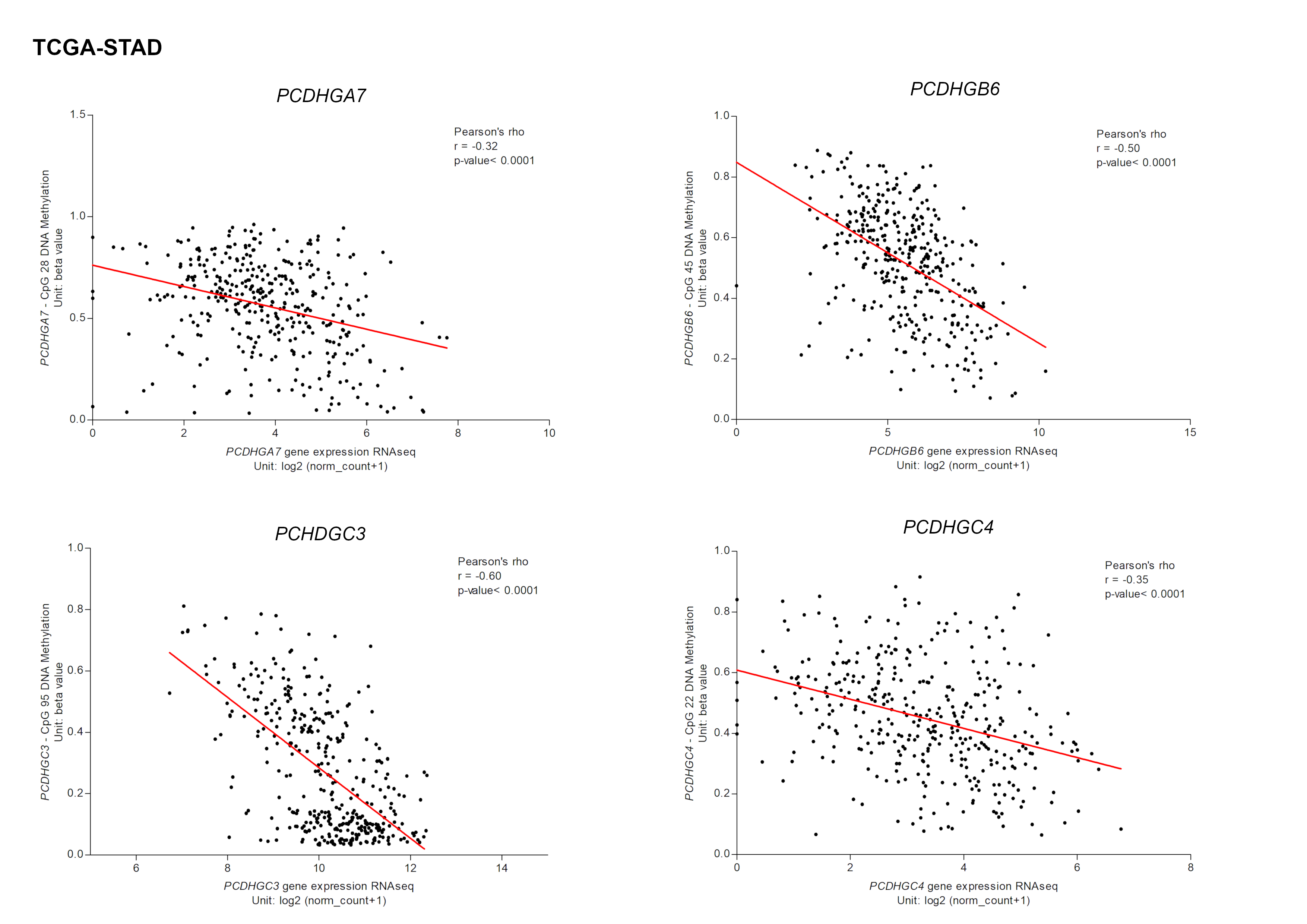

Supplement: Supplementary file 5 — Figure S4. In silico correlation analysis between methylation and expression level of specific PCDHGs in TCGA-STAD. Correlation analysis between the altered CGIs, localized in the promoter region of PCDHGA7, PCDHGB6, PCDHGC3 and PCDHGC4, using the TCGA-STAD dataset. CpG 28, 45, 95 and 22 correspond to UCSC CGI names. (TIFF 1196 kb) [file 13148_2019_695_MOESM5_ESM.tiff]

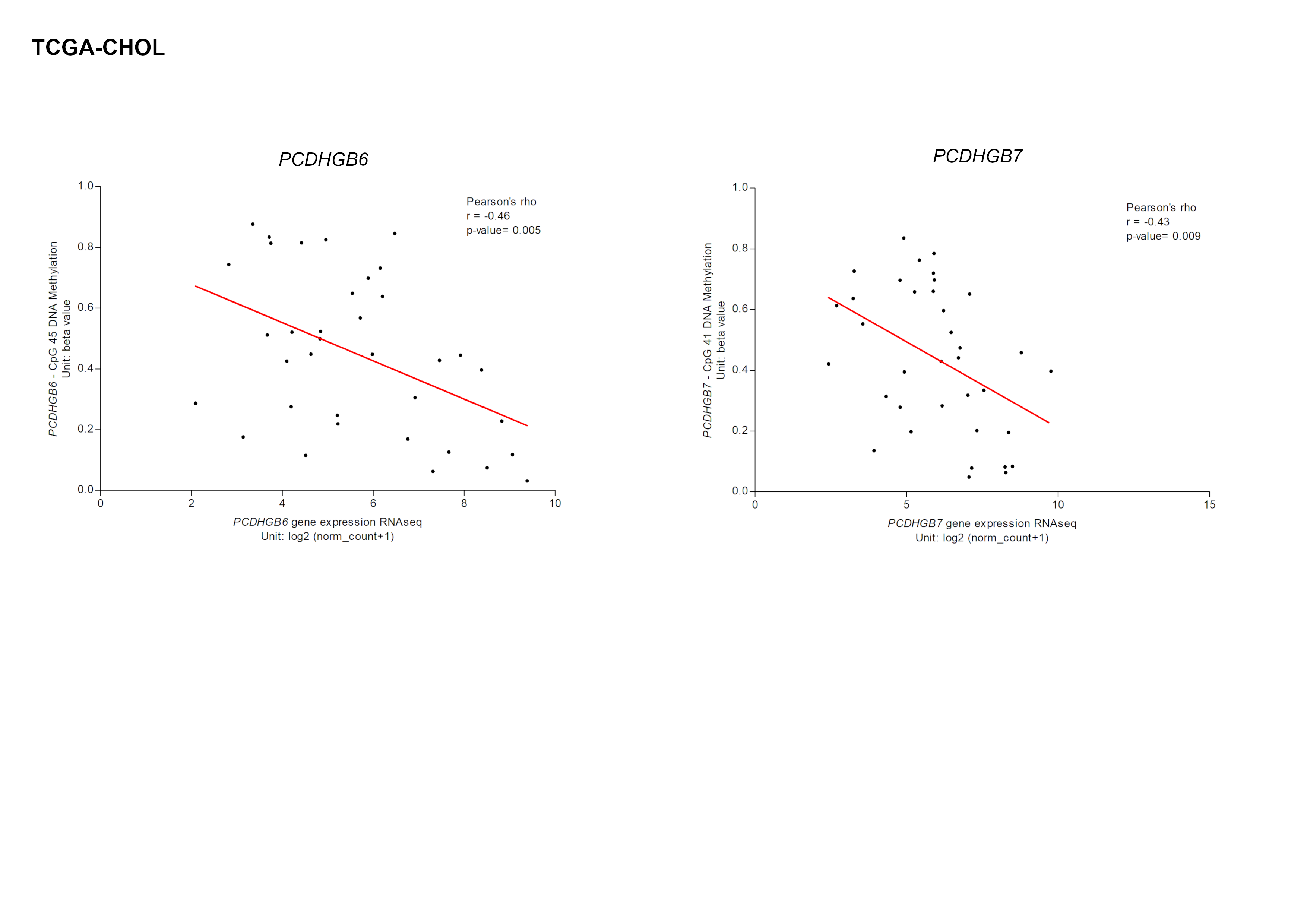

Supplement: Supplementary file 6 — Figure S5. In silico correlation analysis between methylation and expression level of specific PCDHGs in TCGA-CHOL. Correlation analysis between the altered CGIs, localized in the promoter region of PCDHGB6 and PCDHGB7, using the TCGA-CHOL dataset. CpG 45 and 41 correspond to UCSC CGI names. (TIFF 557 kb) [file 13148_2019_695_MOESM6_ESM.tiff]
