# Supplementary material for: Clustered protocadherins methylation alterations in cancer
Source: Clin Epigenetics. 2019 Jul 9;11:100. doi: 10.1186/s13148-019-0695-0 (PMC6617643; doi:10.1186/s13148-019-0695-0)
Supplement: Supplementary file 2 — Table S1. CGIs methylation values in CLL. (DOCX 13 kb) [file 13148_2019_695_MOESM2_ESM.docx]

**Additional file 2**

**Table S1**

**CGIs methylation values in CLL**

| **UCSC CGI** | **UCSC CGI name** | **CTCF binding site** | ***In silico* Δβ value (CLL-Control)** | **Δβ value (CLL-Control)** | **Genes within region** |
| --- | --- | --- | --- | --- | --- |
| chr5:140174573-140174888 | CpG 28 | chr5:140173111-140175577 | 0.189 | 0.158 | PCDHA1, PCDHA2 |
|  |  | chr5:140173795-140176243 |  |  |  |
| chr5:140762401-140762768 | CpG 28 | chr5:140761029-140763470 | 0.135 | 0.163 | PCDHGA1, PCDHGA2, PCDHGA3, PCDHGA4, PCDHGA5, PCDHGA6, PCDHGA7, PCDHGB1, PCDHGB2, PCDHGB3 |
|  |  |  |  |  |  |
| chr5:140180844-140181082 | CpG 21 | chr5:140179374-140181807 | 0.182 | 0.165 | PCDHA1, PCDHA2, PCDHA3 |
|  |  | chr5:140180022-140182456 |  |  |  |
| chr5:140186792-140187268 | CpG 40 | chr5:140185348-140187798 | 0.135 | 0.160 | PCDHA1, PCDHA2, PCDHA3, PCDHA4 |
|  |  | chr5:140186021-140188437 |  |  |  |

*Note*: CGI: CpG island; CLL: chronic lymphocytic leukemia. CpG 28, 21 and 40 correspond to UCSC CGI name.
